# Flexibility and mobility of SARS-CoV-2-related protein structures

**DOI:** 10.1038/s41598-021-82849-2

**Published:** 2021-02-19

**Authors:** Rudolf A. Römer, Navodya S. Römer, A. Katrine Wallis

**Affiliations:** 1 CY Advanced Studies and LPTM (UMR8089 of CNRS), CY Cergy-Paris Université, 95302 Cergy-Pontoise, France; 2 Department of Physics, University of Warwick, Coventry, CV4 7AL UK; 3 School of Life Sciences, University of Lincoln, Brayford Pool Campus, Lincoln, LN6 7TS UK; 4 School of Life Sciences, University of Warwick, Coventry, CV4 7AL UK

**Keywords:** Viral proteins, Biological physics, Biophysics, Computational biology and bioinformatics, Biochemistry, Proteins

## Abstract

The worldwide CoVid-19 pandemic has led to an unprecedented push across the whole of the scientific community to develop a potent antiviral drug and vaccine as soon as possible. Existing academic, governmental and industrial institutions and companies have engaged in large-scale screening of existing drugs, in vitro, in vivo and in silico. Here, we are using in silico modelling of possible SARS-CoV-2 drug targets, as deposited on the Protein Databank (PDB), and ascertain their dynamics, flexibility and rigidity. For example, for the SARS-CoV-2 spike protein—using its complete homo-trimer configuration with 2905 residues—our method identifies a large-scale opening and closing of the S1 subunit through movement of the S${}^\text{B}$ domain. We compute the full structural information of this process, allowing for docking studies with possible drug structures. In a dedicated database, we present similarly detailed results for the further, nearly 300, thus far resolved SARS-CoV-2-related protein structures in the PDB.

## Introduction

At the end of 2019 a cluster of pneumonia cases was discovered in Wuhan city in China, which turned out to be caused by a novel coronavirus, SARS-CoV-2^1^. Since then the virus has spread around the world and currently has caused over 75 million infections with nearly 1.7 million deaths worldwide (Dec 21, 2020)^2^. SARS-CoV-2 is the seventh identified coronavirus that causes human disease. Four of these viruses cause infections similar to the common cold and three, SARS-CoV, MERS-CoV and SARS-CoV-2 cause infections with high mortality rates^3^. As well as affecting humans, coronaviruses also cause a number of infections in farm animals and pets^4^. The risk of future cross-species transmission, especially from bats, has the potential to lead to future pandemics^5^. Thus, there is an urgent need to develop drugs to treat infections and a vaccine to prevent this disease. Some success has already been achieved for SARS-CoV-2 with dexamethasone reducing mortality in hospitalised patients^6^ and a number of vaccine trials are currently ongoing.

The viral spike protein is of particular interest from a drug- and vaccine-development perspective due to its involvement in recognition and fusion of the virus with its host cell. The spike protein is a heavily glycosylated homotrimer, anchored in the viral membrane. It projects from the membrane, giving the virus its characteristic crown-like shape^3^. The ectodomain of each monomer consists of an N-terminal subunit, S1, comprising two domains, S${}^\text{A}$ and S${}^{\text{B}}$, followed by an S2 subunit forming a stalk-like structure. Each monomer has a single membrane-spanning segment and a short C-terminal cytoplasmic tail^7^. The S1 is involved in recognition of the human receptor, angiotensin-converting enzyme 2 (ACE2). This subunit has a closed or down configuration where all the domains pack together with their partners from adjacent polypeptides^8,9^. However, in order for recognition and binding to ACE2 to take place, one of the three S${}^\text{B}$ domains dissociates from its partners and tilts upwards into the open or up configuration^8,9^. Binding of ACE2 to the open conformation leads to proteolytic cleavage of the spike polypeptide between S1 and S2^7^. S2 then promotes fusion with the host cell membrane leading to viral entry^10^. In the following we compute the full, structurally-resolved motion pathway from the open to the closed structure of the full homo-trimer. We will show that in the closed states, the vibrational motion of the trimer closely follows the behavior of an elastic cylinder. In contrast, the most important motion of open structure is overall much more flexible and a particularly high mobility in the S${}^\text{B}$ domains can lead to a full closure of the trimer. The full structural details is this motion pathway are available via our approach. Drugs that target this motion of the spike protein therefore have the potential to prevent infection of host cells.

The main protease of the virus (M${}^\text{pro}$) is another important drug target. M${}^\text{pro}$ is responsible for much of the proteolytic cleavage required to obtain the functional proteins needed for viral replication and transcription. These proteins are synthesised in the form of polyproteins which are cleaved to obtain the mature proteins^11^. M${}^\text{pro}$ is active in its dimeric form but the SARS-CoV M${}^\text{pro}$ is found as a mixture of monomer and dimers in solution^12^. SARS-CoV M${}^\text{pro}$ has been crystallised in different, pH-dependent conformations suggesting flexibility, particularly around the active site. MD simulations support this flexibility^13^. The protease is highly conserved in all coronaviruses, including SARS-CoV-2, so targeting either dimerization or enzymatic activity may give rise to drugs that can target multiple coronaviruses, known and yet unknown^14,15^.

Since the discovery of SARS-CoV-2, a plethora of structures have been determined including M${}^\text{pro}$^16^ and the ectodomain of the spike protein^8,9^ as well as other potential drug and vaccine targets. These structures provide the opportunity for rational drug design using computational biology to identify candidates and optimise lead compounds. However, crystal structures only provide a static picture of proteins, whereas proteins are dynamic. This property is often important in drug development. For example, agonists and antagonists often bind different conformations of G coupled-protein receptors^17^. Flexibility also affects the thermodynamic properties of drug binding^18^, yet the ability to assess flexibility is often hampered by the long computational times needed for MD simulations.

We use a recent protein flexibility modeling approach^19^, combining methods for deconstructing a protein structure into a network of rigid and flexible units (First)^20^ with a method that explores the elastic modes of motion of this network^21–25^, and a geometric modeling of flexible motion (Froda)^26,27^. The usefulness of this approach has recently been shown in a variety of systems^28–32^. Methods similar in spirit, exploring flexible motion using geometric simulations biased along “easy”directions, have also been implemented using Frodan^33^ and NMSim^34^. We have performed our analysis through multiple conformational steps starting from the crystal structures of SARS-CoV-2-related proteins as currently deposited in the PDB. This results in a comprehensive overview of rigidity and the possible flexible motion trajectories of each protein. We emphasize that these trajectories do not represent actual stochastic motion in a thermal bath as a function of time, but rather the possibility of motion along a set of most relevant low-frequency elastic modes. Each trajectory leads to a gradual shift of the protein from the starting structure and this shift may reach an asymptote, where no further motion is possible along the initial vector, as a result of steric constraints^31^. Energies associated with such a trajectory for bonds, angles, electrostatics, and so forth, have be estimated in previous studies of other proteins and shown to be consistent and physically plausible. Computing times for the method vary with the size of the proteins, but range from minutes to a few hours and are certainly much faster than thermodynamically equilibrated MD simulations. Hence, the approach offers the possibility of large-scale screening for protein mobilities.

## Results

### Protein selection

We have downloaded protein structure files as deposited on the Protein Data Bank^35^, including all PDB codes that came up when using “SARS-CoV-2” and “Covid-19” as search terms, as well as minor variations in spelling. In our first search of April 18th, 2020, this resulted in 133 protein structures. Further searches on May 20th and 29th, and August 25th, 2020 gave a total of 287 structures. Many of the structures found, as outlined above, have been deposited on the PDB in dimer or trimer forms. Hence one has the choice to study the rigidity and motion of the monomer or the dimer/trimer. Clearly, the computational effort for a dimer/trimer structure is much larger than for a monomer since, in addition to the intra-monomer bond network, inter-monomer bonds also need to be taken into account. Furthermore, it is not necessarily clear whether the possible motion of a monomer or dimer should be computed to have the most biological relevance. We have calculated the motion of full dimer and trimer structures only for a few selected and biologically most relevant such structures while the default results concentrated on the monomers. Nevertheless, we wish to emphasize that when results for a certain monomer exist, it should nearly always be possible to also obtain their motion in dimer/trimer configuration. For some protein structures, we have found that steric clashes were present in the PDB structures that made a flexibility and sometimes even just the rigidity analysis impossible. Usually, this is due to a low crystal resolution. A list of all current protein PDB IDs included in this work is given in Table S1.

### Rigidity and flexibility

**Figure 1:**
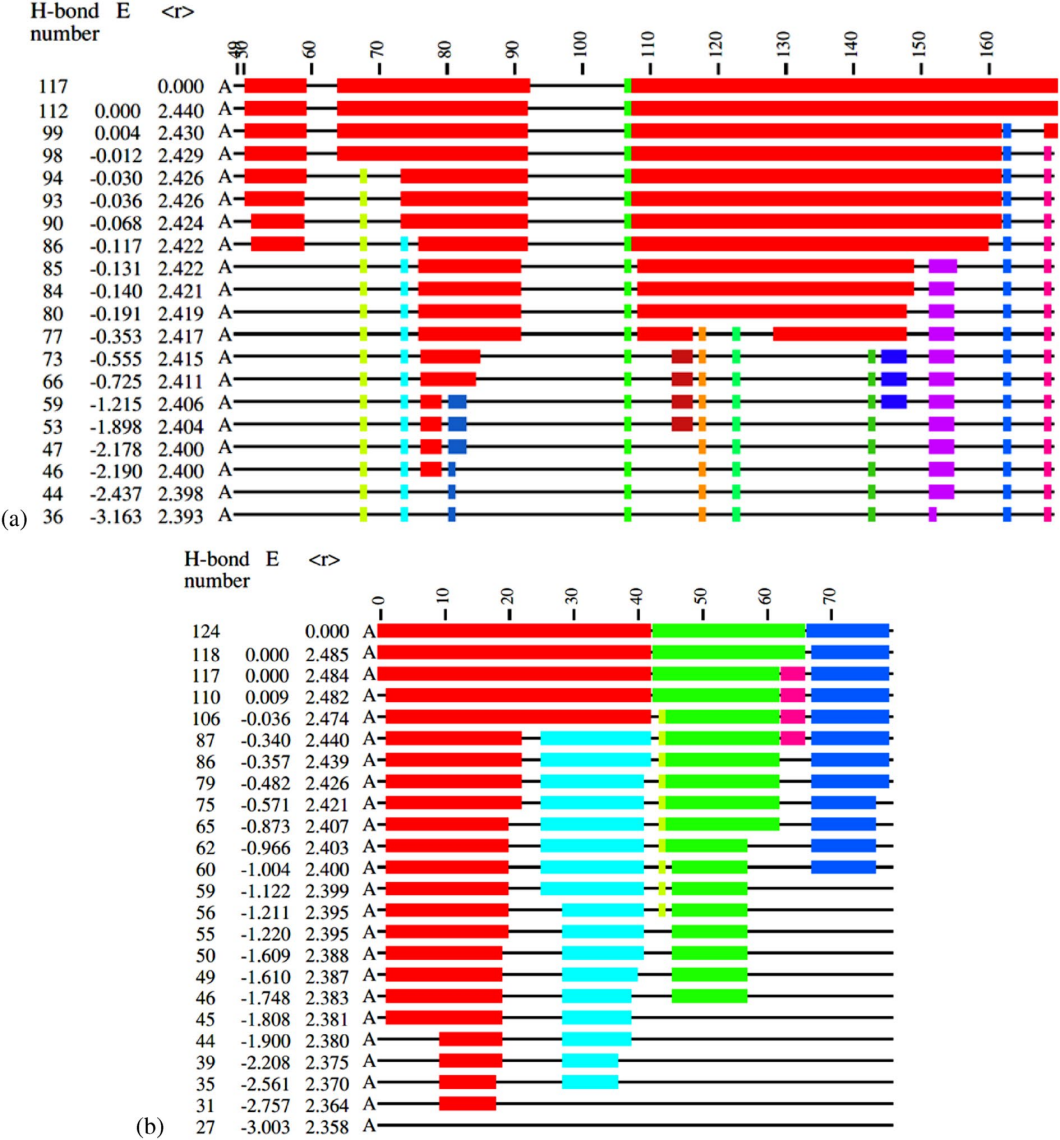
Examples of rigid cluster decompositions for different SARS-CoV-2 protein structures. (**a**) Shows the cluster decomposition for the crystal structure of the SARS-CoV-2 nucleocapsid protein N-terminal RNA binding domain (PDB: 6m3m) while (**b**) gives chain A of the co-factor complex of NSP7 and the C-terminal domain of NSP8 from SARS CoV-2 (PDB: 6wiq). Different rigid clusters of the polypeptide chain appear as coloured blocks along the protein chain with each $C_{\alpha }$ labelled from its N-terminal at 1 to its C-terminal. The three columns at the left of each cluster decomposition denote the index of the hydrogen bond to have been opened last (with 117 and 124 to total number of *H* bonds in (**a**) and (**b**), respectively), the energy cutoff $E_\text {cut}$ (E) and the mean number of bonded neighbours $\langle r \rangle$ (mean coordination) at that value of $E_\text {cut}$. The label A indicates that protein chain A is being considered from the structure. We emphasize that the $E_\text {cut}$ scale is not linear since new rows are only added when the rigidity of a structure has changed. When the value of $E_\text {cut}$ decreases (the downward direction points towards larger, negative $E_\text {cut}$ magnitudes), rigid clusters break up and flexibility increases. The colour coding shows which atoms belong to which rigid cluster. Flexible regions appear as thin black lines.

**Figure 2:**
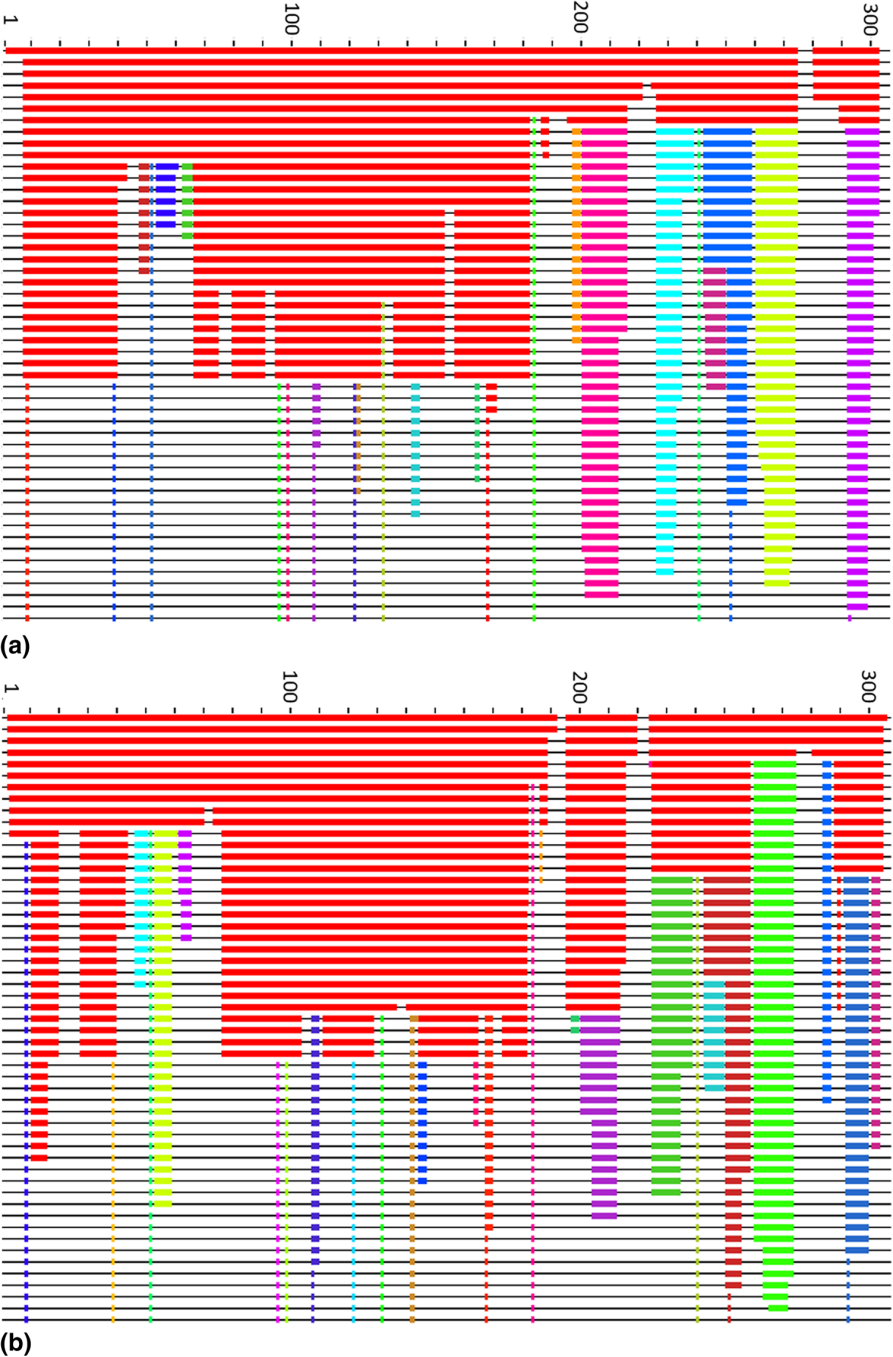
Examples of rigid cluster decompositions for different SARS-CoV-2 protein structures. (**a**) Shows the monomer of the crystal structure of the COVID-19 main protease in apo form (PDB: 6m03) and (**b**) gives results for chain A of a complex resulting from the reaction between the SARS-CoV main protease and a carbamate (PDB: 6y7m). Distinct rigid clusters of the polypeptide chain appear as coloured blocks along the protein chain with each $C_{\alpha }$ labelled from its N-terminal at 1 (left edge) to its C-terminal (right edge). The coloring and choice of blocks follows the same logic as detailed in Fig. 1 except that the left most data columns are not shown for clarity. The energy cutoff $E_\text {cut}$ decreases in the downward direction towards larger, more negative $E_\text {cut}$ magnitudes; rigid clusters break up and more of the chain becomes flexible.

In Figs. 1 and 2 we show examples of different rigidity patterns that emerge from the First analysis. In line with previous studies comparing these rigidity patterns across various protein families^19^, we find that they can be classified into about four types. In Fig. 1a we see that for the crystal structure of SARS-CoV-2 nucleocapsid protein N-terminal RNA binding domain (PDB: 6m3m), the largest rigid cluster in the pristine structure, i.e. at $E_\text {cut}=0$, largely remains rigid through the dilution process of consecutively lowering $E_\text {cut}$ values. When bonds are opened the rigid cluster shrinks but the newly independent parts are flexible and not part of any new large rigid cluster themselves. Only very small parts of the protein chain break to form their own independent rigid structures before at a certain $E_\text {cut}$ the whole protein is essentially flexible. We shall denote such a behaviour as *brick*-like. In contrast, in Fig. 1b we observe that for chain A of the co-factor complex of NSP7 and the C-terminal domain of NSP8, which forms part of the RNA synthesis machinery from SARS CoV-2 (PDB: 6wiq), already the crystal structure has fallen into three independent rigid structures. Opening bonds, we find that the largest rigid cluster breaks into 2 rigid structures. Then these now four *domains* retain their character upon opening more and more bonds until they simply dissolve into full flexibility (We use the term *domain* here to denote large regions of a protein connected in the same rigidity cluster. Usually, domains in a biological sense are also detected as domains in a rigidity sense as used here^32^. However, this relationship is not necessarily always the case.).

While brick- and domain-like behaviour is only found occasionally, two further types of rigidity dissolution are more prevalent. In Fig. 2a we see that for, e.g. the monomer of the crystal structure of the COVID-19 main protease (M${}^\text{pro}$) in apo form (PDB: 6m03), the rigid cluster dominating the crystal structure quickly falls apart upon change in $E_\text {cut}$ with five newly formed independent rigid clusters emerging towards the C-terminal of the protein chain. These new clusters remain stable to the opening of further bonds, even when the remnants of the original rigid cluster has become fully flexible. Such a behaviour relies on a certain critical $E_\text {cut}$ value to dominate the rigidity dissolution and is reminiscent of so-called first-order phase transitions in statistical physics. Hence this rigidity-type is usually denoted as *1st order*^19^. The behaviour seen in 2b for the crystal structure of the complex resulting from the reaction between the SARS-CoV main protease and tert-butyl (1-((S)-3-cyclohexyl-1-(((S)-4-(cyclopropylamino)-3,4-dioxo-1-((S)-2-oxopyrrolidin-3-yl)butan-2-yl)amino)-1-oxopropan-2-yl)-2-oxo-1,2-dihydropyridin-3-yl)carbamate (PDB: 6y7m) is markedly different. Here, there are many values of $E_\text {cut}$ where large parts of the original rigid structure break off one after another so that towards the end of the bond-opening process, the original cluster is still present, but no longer dominates the rigidity pattern. This behaviour is called *2nd order*^19^.

In Table S1, we have indicated the classification for each protein structure into the four classes. Obviously, this classification is not perfect and there are also sometimes intermediate rigidity patterns. Nevertheless, this rigidity classification already provides a first insight into the possible flexibility and range of motion for each structure. It should be clear, that a brick-like rigidity shows the least flexibility until it dissolves completely. On the other hand, for domain-like structures, one can expect possible intra-domain motion while inter-domain motion might be harder to spot. Similarly, for a 1st-order rigidity, one would expect little dynamic mobility until the “transition” value for $E_\text {cut}$ has been reached, although high levels of flexibility should be possible afterwards. Lastly, a protein with 2nd-order rigidity should have the most complex behaviour in terms of flexibility since new possible mobility can be expected throughout the range of $E_\text {cut}$ values.

### Protein mobility

**Figure 3:**
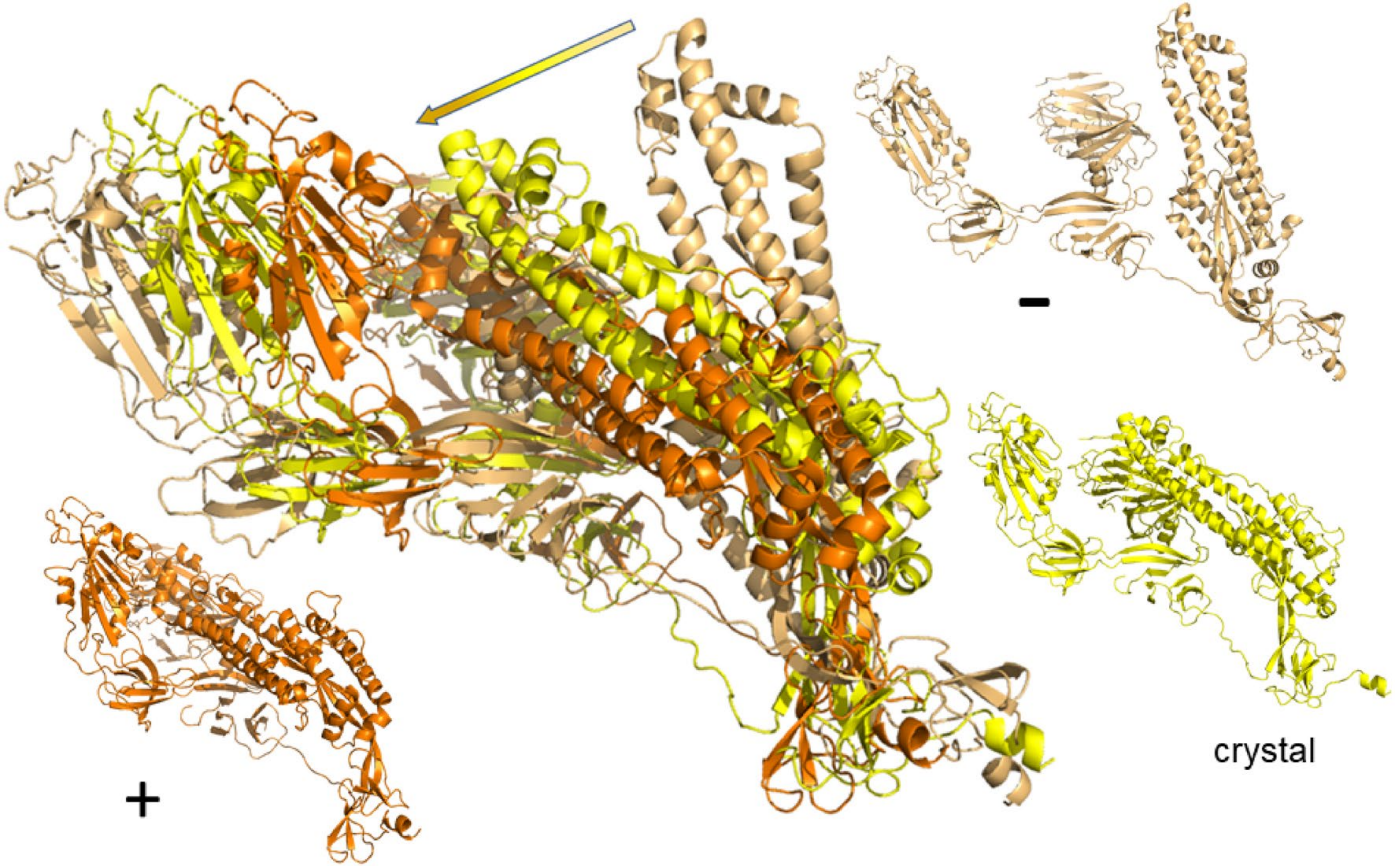
Example of flexible motion for mode $m_7$ of the SARS-CoV-2 spike ecto domain (PDB: 6vxx) monomer at $E_\text {cut}={0.5}\;{\text {kcal/mol}}$ with chain A denoted by yellow with its extreme structural positions superimposed and colored light orange/orange. The arrow indicates the range of movement for the dominant $\alpha$-helix structure in the spike ecto domain, from negative to positive movement. The smaller protein structures surrounding the large one show the initial (crystal) structure as well as the extremes ($+$, –) along the possible motion directions for $m_7$ in the same colors as in the superimposed structure.

**Figure 4:**
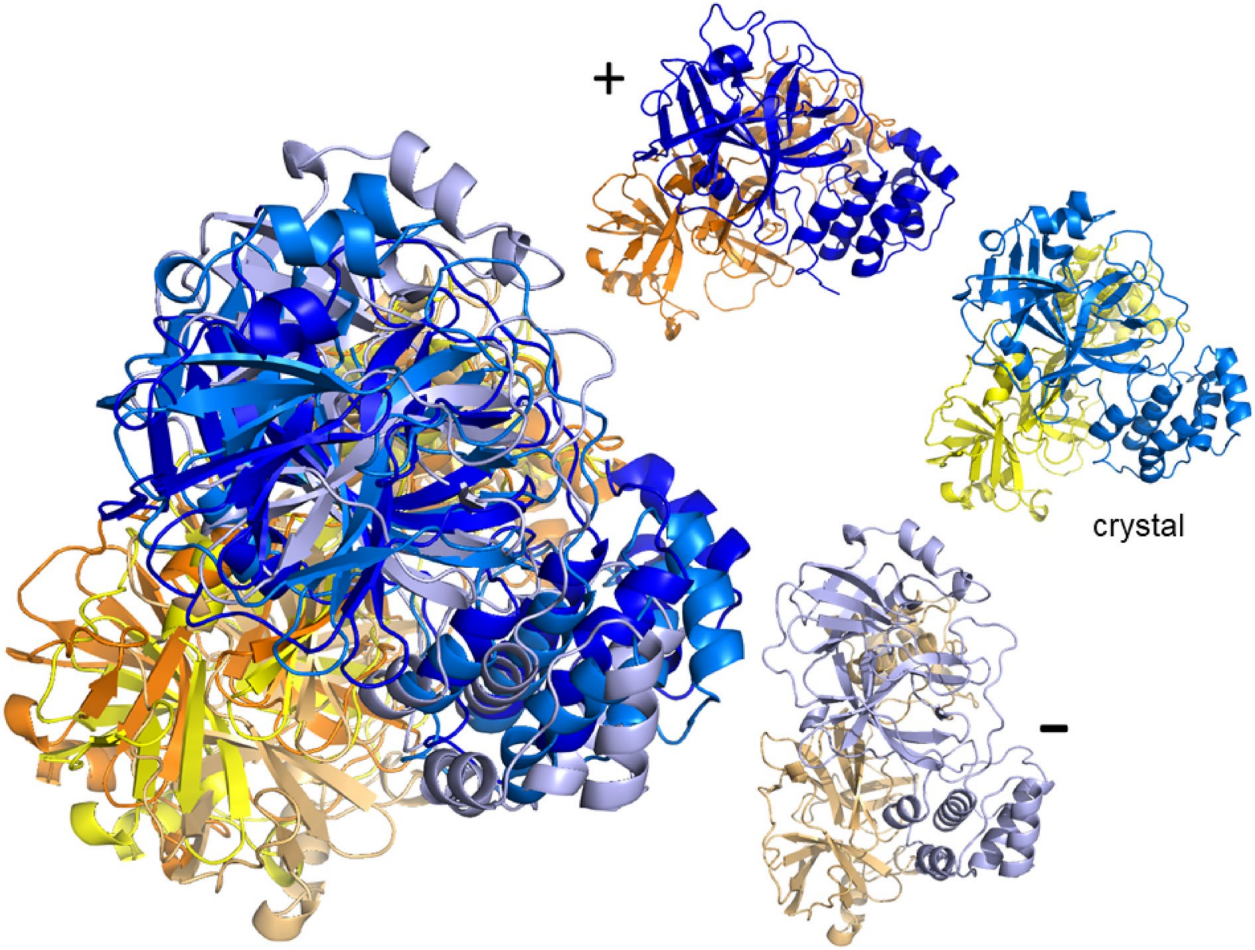
Example of flexible motion for mode $m_7$ of the dimer of main protease (PDB: 6lu7) at $E_\text {cut}={2}\;{\text {kcal/mol}}$. The crystal structure of the dimer is superimposed with its extreme structural positions according to mode $m_7$. Chain A is denoted yellow and its extremal structures colored as in Fig. 3 light orange/orange; chain B is indicated in blue with extremal positions in light and dark blue. The smaller protein structures show the initial (crystal) structure as well as the extremes ($+$, –) along the possible motion directions for $m_7$ in the same colors as in the superimposed structure.

For each value of $E_\text {cut}$, the First analysis has provided us with a map of rigid and flexible regions in a given crystal structure. We can now translate this into propensity of motion by allowing the flexible parts to move perturbatively, subject to full bonding and steric constraints (see “Methods” section). Moving along directions proposed by an elastic normal model analysis of the crystal structure, we can therefore construct possible motion trajectories that are fully consistent with the bond network and steric constraints. Each trajectory corresponds to one such normal mode, denoted $m_7$ up to $m_{12}$ for the first low-frequency non-trivial such modes, as well as the chosen $E_\text {cut}$ value. Generally, a larger value of $|E_\text {cut}|$ implies less rigidity and results in larger scale flexible motion. In Figs. 3 and 4 we give examples of such motion trajectories. Figure 3 shows a monomer of the SARS-CoV-2 spike glycoprotein (closed state) (PDB code: 6vxx^9^). We can see that there is a good range of motion from the crystal structure when following the normal mode modes into either positive or negative changes along the mode vector. Figure 4 shows the motion for the dimer structure of the SARS-CoV-2 main protease (PDB code: 6lu7, in complex with inhibitor N3. We have removed the inhibitor in the motion analysis here)^16^. Again, one can see considerable motion, although due to the complexity of the structure, it is difficult to distinguish individual movement patterns from such a frozen image. Much better insight can be gained when watching for full range of motion as a movie. Direct access to the movie for the spike ecto domain monomer (6vxx) is provided via Ref.^36^, while the motion of the main protease dimer can be viewed at Ref.^37^. We have made a dedicated web download page^38^ where movies for the complete set of PDB codes as given in Table S1 are publicly available. The movies are being offered for various modes $m_7$ to $m_{12}$ as well as $E_\text {cut}$ values, at least containing results for $E_\text {cut}={-1}\;{\text {kcal/mol}}$, ${-2}\;{\text {kcal/mol}}$ and ${-3}\;{\text {kcal/mol}}$, respectively. In addition, the site includes the rigidity resolutions discussed above and all intermediate structures needed to make the movies. This allows for the calculation of relative distances and other such structural measures along each motion trajectory as desired. For a detailed analysis of the SARS-CoV-2 main protease with similar methods, we refer the reader to Ref.^39^.

### SARS-CoV-2 spike ecto domain structures

The trans-membrane spike glycoprotein mediates entry into host cells^8,9^. As such, it is a “main target for neutralizing antibodies upon infection and the focus of therapeutic and vaccine design”^9^. It forms homotrimers protruding from the viral surface^3^. Up to the end of May 2020, 3 structures of the trans-membrane spike glycoprotein have been deposited in the PDB. These transmission electron cryomicroscopy (cryo-EM) studies have led to structures with PDB codes 6vsb^8^, 6vxx^9^ and 6vyb^9^ now being available. With RMS resolution of 3.5 Å, 6vsb has a slightly lower resolution than 6vxx at 2.8 Å and 6byv at 3.2 Å. In the following, we shall discuss the resulting rigidity and flexibility properties of these three structures in their full trimer form. Results for individual monomer rigidities, as given in Fig. 3, are also available at Ref.^﻿38^.

#### Prefusion SARS-CoV-2 spike glycoprotein with a single receptor-binding domain open

The rigidity pattern of the homotrimer for this structure (PDB: 6vsb, ${440.69}\;{\text {kDa}}$, 22854 atoms, 2905 residues) is dominated by a large rigid cluster encompassing most of the trimer structure except for a region from roughly residue 330 to residue 525 (With nearly 3000 residues in the structure of the trimer, is it impossible to capture the behaviour in a single figure that at the same time would allow the reader to see enough detail. The reader is referred to Ref.^38^ for access to the electronic images.). This corresponds to the S${}^\text{B}$ domain of S1 in each monomer. In the trimer configuration, it is known that one of the S${}^\text{B}$ domains can change from the closed to an open configuration^8^. At $|E_\text {cut}|={0.016}\;{\text {kcal/mol}}$, the 6vsb structure of the trimer breaks into many different rigid parts, but the original large cluster remains a dominating feature across the rigidity dilution plot. A motion analysis does not compute but breaks with bad sterics, apparently due to the comparably low resolution of the crystal structure.

#### Mobility of the SARS-CoV-2 spike glycoprotein (closed state)

**Figure 5:**
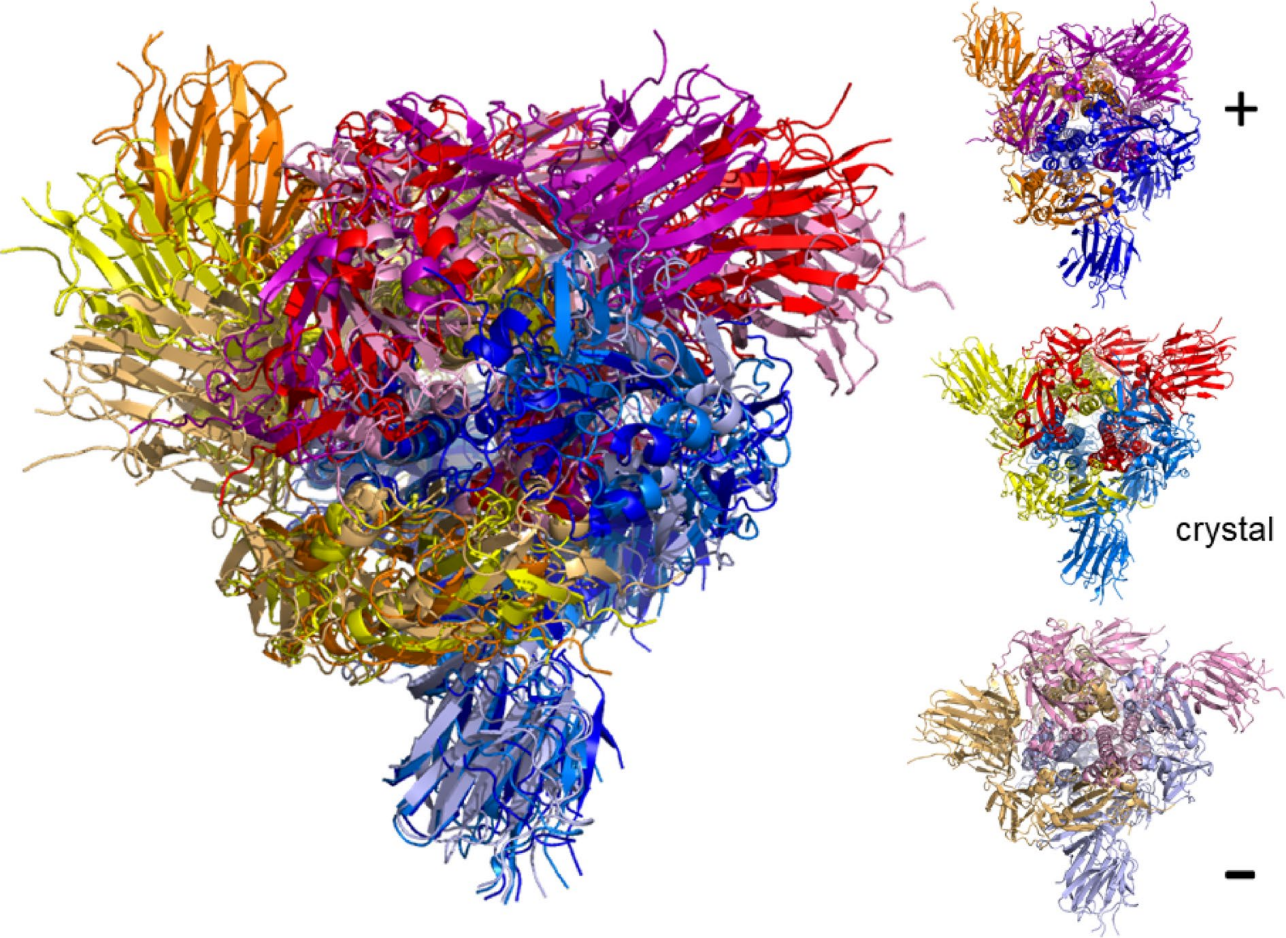
Top view of the spike ecto domain in the *closed* configuration (6vxx)^9^ for mode $m_7$ motion at $E_\text {cut}={2}\;{\text {kcal/mol}}$ for the whole trimer. The secondary protein structure is highlighted by the chosen “cartoon” representation. Colors yellow, blue and red denote chains A, B, and C, respectively, while color combinations light orange/orange, pink/purple and light blue/dark blue show the extreme structural positions for the movements along the normal mode $m_7$ at $E_\text {cut}={2}\;{\text {kcal/mol}}$. As in Fig. 3, the smaller protein structures show the initial (crystal) structure as well as the extremes ($+$, –) along the possible motion directions for $m_7$ in the same colors as in the superimposed structure.

**Figure 6:**
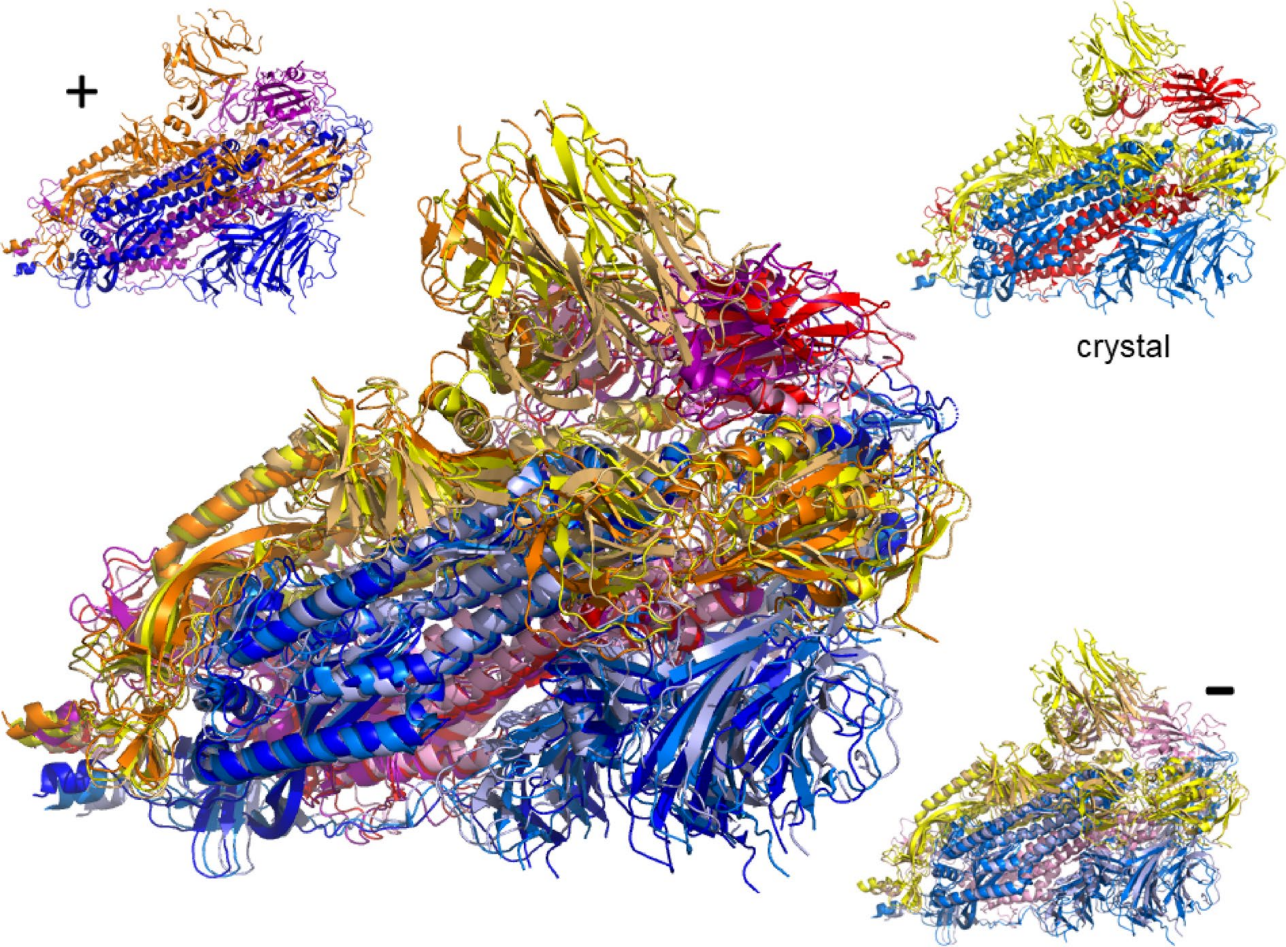
Side views of motion in the spike ecto domain in *closed* configuration (6vxx)^9^ for $m_7$ motion at $E_\text {cut}={2}\;{\text {kcal/mol}}$ for the whole trimer. The secondary protein structure is highlighted by the chosen “cartoon” representation. As in Fig. 5, colors yellow, blue and red denote chains A, B, and C, respectively, while color combinations light orange/orange, pink/purple and light blue/dark blue show the extreme structural positions for the movements along $m_7$. The smaller protein structures show the initial (crystal) structure as well as the extremes ($+$, –) along $m_7$ in the same colors as in the superimposed structure.

For the closed state structure (6vxx, ${438.53}\;{\text {kDa}}$, Atom Count: 23694 atoms, 2916 residues) of the SARS-CoV-2 spike glycoprotein, the rigidity pattern is again “brick”-like and the whole of the crystal structure is part of a single cluster. At $|E_\text {cut}|={0.022}\;{\text {kcal/mol}}$, there is a first order break of the large rigid cluster into dozens of smaller rigid units. Nevertheless, the original cluster retains a good presence in each chain of the trimer. We are now able to produce motion studies for the full set of modes $m_7$ to $m_{12}$ and various $E_\text {cut}$’s. In Figs. 5 and 6 we show the results, again using the structures for the extreme ranges of the motion as in Fig. 3. Figure 6 shows a view from the top while Fig. 5 gives a view from the side of the spike protein. Both views are chosen similar to those given in Ref.^9^. The movies corresponding to Figs. 5 and 6 can be viewed at Refs.^40,41^, respectively. Looking at the whole range of modes and $E_\text {cut}$’s computed, we find that the motion is very reminiscent of the vibrational excitations of a rigid cone or cylinder. There is twist motion around the central axis, bending of the trimer along the central axis, relative twist motion of 2 chains relative to the remaining chain (c.p. Fig. 5), etc. At $m_{12}$ (with $|E_\text {cut}|={2}\;{\text {kcal/mol}})$, large scale motion has already stopped and one only observes smaller scale motion in flexible parts of the trimer chains. Overall, this behaviour is very consistent with the elastic behaviour of a “closed” structure^9^ such as a cone or cylinder.

#### Mobility of the SARS-CoV-2 spike glycoprotein (open state)

**Figure 7:**
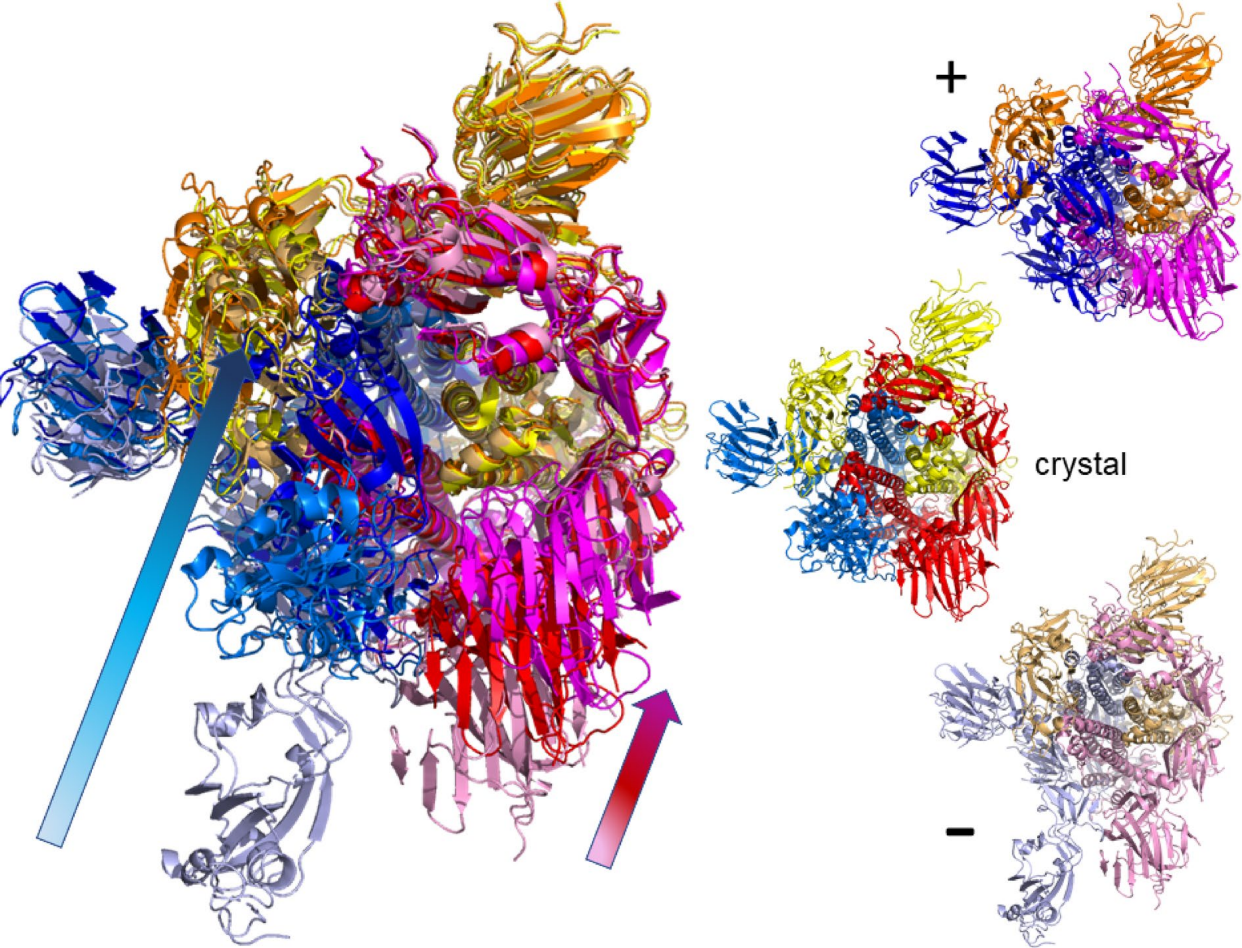
Possible motion along mode $m_7$ at $E_\text {cut}={1}\;{\text {kcal/mol}}$ in the *open* spike ecto domain (6vyb)^9^ when in view from the top. Colors for the superimposed structure are chosen identical to Figs. 5 and 6. As in Fig. 3, the arrows indicate the range of motion for chain B (blue shades) and also for chain C (reds). The initial (crystal) structure as well as the extremes ($+$, –) along $m_7$ in the same colors as in the superimposed structure are shown in the three smaller proteins.

**Figure 8:**
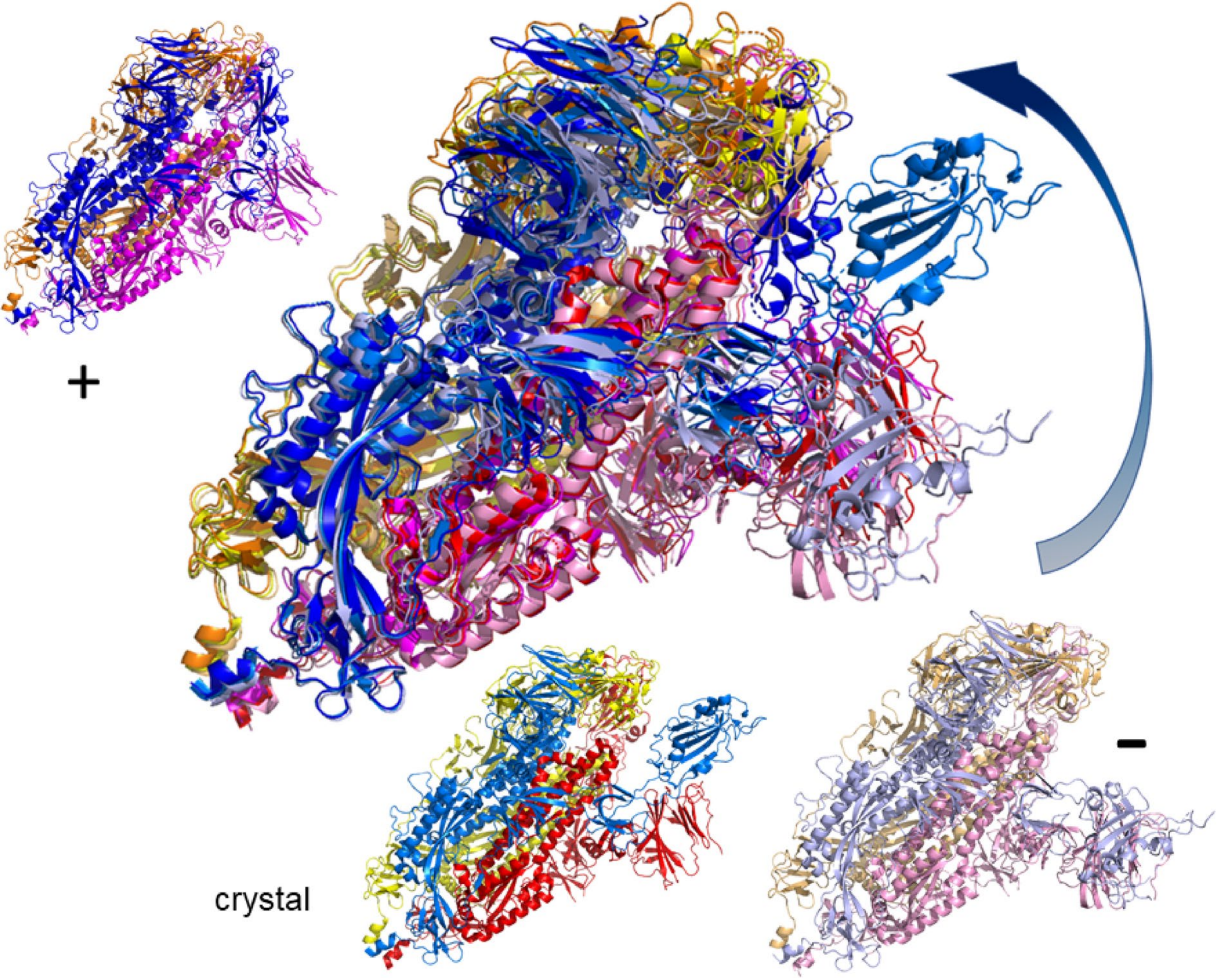
Possible motion along mode $m_7$ at $E_\text {cut}={1}\;{\text {kcal/mol}}$ in the *open* spike ecto domain (6vyb)^9^ in side view. Colors are chosen identical to Fig. 5. As in Figs. 3 and 7, the arrow shows the range of motion for chain B (blue shades). The initial (crystal) structure as well as the extremes ($+$, –) along $m_7$ are shown in the three smaller proteins, using the same colors as in the superimposed structure.

We now turn to the last SARS-CoV-2 spike ecto domain structure (6vyb, ${437.65}\;{\text {kDa}}$, 22365 atoms, 2875 residues)^9^. This structure is in the open configuration similar to the conformation seen in 6vsb^8^. The structure is shown in top view in Fig. 7 and in side view in Fig. 8. From the rigidity plots we find, in addition to the largest rigid cluster, already for the crystal structure at $|E_\text {cut}|={0}\;{\text {kcal/mol}}$ a second rigid cluster, also roughly spanning from residue 330 to 525. Again, this region identifies the S${}^\text{B}$ domain as in the 6vsb structure. Compared to the closed state (6vxx), we see that this cluster has more internal structure, i.e. consists of more flexible parts in the S${}^\text{B}$ region from 330 to 525 and also seems to fall apart upon further increasing the $|E_\text {cut}|$ value. This suggests that it is more flexible as a whole. Upon motion simulation, see^42,43^ for side and top views, we observe a very high mobility in that prominent S${}^\text{B}$ subdomain. Starting from the crystal structure, we find for $|E_\text {cut}|={1}\;{\text {kcal/mol}}$ a clear further opening towards a negative Froda mode, $m_7$, while in the other, positive direction of $m_7$, the structure can again close the trimer. The distance range of the motion can be expressed as follows: the distance from residue 501 in the middle of the central $\beta$-sheet of the S${}^\text{B}$ to the most opened conformation is 57 Å while distance to the same residue in the most closed conformation is 28 Å. The distance from open to closed is 69 Å. Hence the motion simulation adds additional insight into the distinction between the open (6vyb) and the closed (6vxx) structures while also showing that a transition from open to closed is indeed possible. As stated in Ref.^9^, this interplay of closing and opening is expected to be central to the viral entry into the human cell.

### Other SARS-CoV-2 structures of interest

Similar results have been obtained^38^ for the other about 300 Covid-19 structures thus far deposited in the PDB. For example, from those structures deposited from late May to August 2020, the nsp7-nsp8c complex (PDB code: 6m5i)^44^ is potentially interesting as this complex is part of the RNA transcription and replication machinery. The motion of the hetero dimer shows an intriguing movement restricted to just one of the total seven $\alpha$ helices. The motion of ORF3a (PDB code: 6xdc)^45^, a putative ion channel, might be important in understanding the inflammatory response resulting from Covid-19 infection, i.e. as deletion has been shown to reduce viral titer. We find considerable flexibility in the homo dimer structure between the $\alpha$-helix-rich and the $\beta$-sheet regions. On the other hand, SARS-CoV-2 Orf9b, which potentially suppresses the interferon response upon infection leading to a less effective immune response^46^, shows very little flexibility (PDB code: 6z4u)^47^.

## Discussion

SARS-CoV-2 infectivity is dependent on binding of the spike protein to ACE2. This binding is only possible when the spike protein is in the open conformation. Structures of both the open and closed conformation have already been determined and the flexibility of the S${}^\text{B}$ domain inferred from these static structures^8,9^. The spike trimer consists of almost 3000 amino acids; it not an easy target for dynamics simulations due to its size. In the open structures (6vsb and 6vyb) the S${}^\text{B}$ domain is easily identifiable in the rigidity analysis as a separate cluster to the rest of the trimer. This shows that the domain has increased flexibility. We can clearly observe the hinge movement of the S${}^\text{B}$ domain in the open configuration (6vyb), with the S${}^\text{B}$ domain moving back into the closed configuration. The range of movement from the most opened to the most closed conformation can be measured to be quite large. The flexibility within the S${}^\text{B}$ domain itself during the hinge movement is also seen to be considerable. All these findings suggest that the S${}^\text{B}$ domain of the spike protein has the necessary flexibility to attach itself readily to ACE2. However, when starting from the closed structure (6vxx), we do not see an opening. This suggests possibly stronger bonds and steric constraints which need to be overcome before the structure is able to open up. Nevertheless, to our knowledge this is the first time the hinge motion of the S${}^\text{B}$ domain has been predicted solely based on the dynamics of a structure. In principle, the full structural information provided in our download site^38^ can be used to perform possible docking studies with ligands of different sizes. These intermediate conformations can also be used as convenient starting points for further MD-based dynamics studies to evaluate thermodynamic properties of the structures. This shows the power of FIRST and FRODA to complement structure determinations and MD simulations to make valid predictions about the dynamics of proteins. Similarly detailed structural analyses and further MD work is also possible for the other structures included in this work. The classification of the protein structures into four types of dynamics provides information about the level of flexibility of a protein which is relevant to drug discovery. For more detailed information rigidity dilution plots and motion movies are available for download at Ref.^38^. In addition, the code underlying our study is accessible at Ref.^48^ and implemented in a free web server at Ref.^49^.

## Methods

### Rigidity analysis

In each case we start the rigidity, flexibility and mobility modelling with a given protein crystal structure file in PDB *.pdb* format. Hydrogen atoms absent from the PDB crystal structures are added using the software Reduce^50^. Alternate conformations that might be present in the protein structure file are removed if needed and the hydrogen atoms are renumbered in PyMol^51^. We find that for some protein structures the addition of hydrogen atoms is not possible without steric clashes. Consequently, identification of a viable structure and its continued analysis is not possible. These proteins are labelled in Table S1. We observe five structures which exhibit such steric clashes.

For the remaining proteins we produce the “rigidity dilution” or rigid cluster decomposition (RCD) plot using First^20^. The plots show the dependence of the protein rigidity on an energy cutoff parameter, $E_\text {cut}< 0$. It parametrizes a bonding range cutoff based on a Mayo potential^52,53^ such that larger (negative) values of $E_\text {cut}$ correspond to more open bonds, i.e. a smaller set of hydrogen bonds to be included in the rigidity analysis.

### Elastic network modes

We obtain the normal modes of motion using elastic network modelling (ENM)^54^ implemented in the ElNemo software^23,24^. This generates a set of elastic eigenmodes and associated eigenfrequencies for each protein. The low-frequency modes are expected to have the largest motion amplitudes and thus be most significant for large conformational changes. The six lowest-frequency modes (modes 1–6) are just trivial combinations of rigid-body translations and rotations of the entire protein. Here we consider the six lowest-frequency non-trivial modes, that is, modes 7–12 for each protein. We denote these modes as $m_7$, $m_8$, $\ldots$, $m_{12}$.

### Mobility simulations

The modes are next used as starting directions for a geometric simulation, implemented in the Froda module^26^ within First. This explores the flexible motion available to a protein within a given pattern of rigidity and flexibility. Froda then reapplies bonding and steric constraints to produce an acceptable new conformation. Since the displacement from one conformation to the next is typically small, we only record every 50th conformation. Typically the computation continues for several thousand conformations. A mode run is considered complete when no further projection along the mode eigenvector is possible (due to steric clashes or bonding constraints). Such a situation manifests itself in slow generation of new conformations. We have performed a Froda mobility simulation for each protein at several selected values of $E_\text {cut}$. This allows us to study each protein at varying stages of its bond network, roughly corresponding to changing environmental conditions, such as different temperatures as well as changes in the solution environments. In a previous publication, we discussed the criteria for a robust selection of $E_\text {cut}$^53^. Ideally, for each protein structure, a bespoke set of $E_\text {cut}$ values should be found, with the RCD plots providing good guidance on which $E_\text {cut}$ values to select. Clearly, for a large-scale study as presented here, this is not readily possible due to time constraints. Instead, we have chosen $E_\text {cut}= {-1}\;{\text {kcal/mol}}, {-2}\;{\text {kcal/mol}}$ and ${-3}\;{\text {kcal/mol}}$ for each protein. These values have been used before in a multi-domain protein with ${22}\;{\text {kDa}}$ and shown to reproduce well the behaviour of (i) a mostly rigid protein at $E_\text {cut}={-1}\;{\text {kcal/mol}}$, (ii) a protein with large flexible substructures/domains at $E_\text {cut}={-2}\;{\text {kcal/mol}}$ and (iii) a protein with mostly flexible parts connecting smaller sized rigid subunits at $E_\text {cut}={-3}\;{\text {kcal/mol}}$^31,53^. In addition, we have also performed the analysis at other values of $E_\text {cut}$ when upon inspection of the RCD plots it was seen that the standard values (i)–(iii) would not be sufficient. The exact values used are given in Table S1.

We emphasize that these trajectories do not represent actual stochastic motion in a thermal bath as a function of time, but rather the *possibility of motion* along the most relevant elastic modes. Each trajectory leads to a gradual shift of the protein from the starting structure. This shift eventually reaches an asymptote, where no further motion is possible along the initial vector, as a result of steric constraints. Energies associated with such a trajectory for bonds, angles, electrostatics, and so forth, can be estimated and shown to be consistent and physically plausible^31^.

## Supplementary Information

Supplementary Information.
