# Supplementary material for: Flexibility and mobility of SARS-CoV-2-related protein structures
Source: Sci Rep. 2021 Feb 19;11:4257. doi: 10.1038/s41598-021-82849-2 (PMC7896093; doi:10.1038/s41598-021-82849-2)
Supplement: Supplementary file 1 — Supplementary Information. [file 41598_2021_82849_MOESM1_ESM.pdf]

Supplemental Material for
Flexibility and Mobility of SARS-CoV-2-related protein structures

Rudolf A. Römer[1,2,3*], Navodya S. Römer[4], and A. Katrine Wallis[5]

[1]CY Advanced Studies and LPTM (UMR8089 of CNRS), CY Cergy-Paris Université, F-95302 Cergy-Pontoise, France
[2]Department of Physics, University of Warwick, Coventry, CV4 7AL, United Kingdom
[4]School of Life Sciences, University of Lincoln, Brayford Pool Campus, Lincoln, LN6 7TS, United Kingdom
[5]School of Life Sciences, University of Warwick, Coventry, CV4 7AL, United Kingdom
[*]r.roemer@warwick.ac.uk

ABSTRACT

The supplemental material contains a list of all PDB structures used as well as links to some of the most important movie representations of the computed motions.

1 Sources and video links

In Table S1 we give the complete list of all PDB codes used in our study, link their supporting publications as well as the list of modes and E_{cut} values used in computing the full motion movies available from Ref.[1]. We also provide the full motion movies for the structures given in the main text in Fig. 3, namely the monomer of the SARS-Cov-2 spike ecto domain (6vxx)[2] and the dimer of the main protease[3]. Similarly, for main text Fig. 4 we include the movies of the closed SARS-Cov-2 spike ecto domain trimer in side[4] and top view[5]. Last, for the open SARS-Cov-2 spike ecto domain trimer in main text Fig. 5, we again give the side[6] and top views[7]. In addition, the rigidity dilution plots are given for the main protease (6lu7[8]), as well as the open and closed spike ecto domains, 6vxx[9] and 6vyb[10], respectively.

PDB code	Ref.	rigidity class.	E_{cut}'s (kcal/mol)	protein type	role	oligomeric state	error
The following PDB files were downloaded 18 April 2020							
5r7y	[11]	1st	1, 2, 3, 0.2	Main protease	cleavage of poly protein	dimer	
5r7z	[11]	1st	1, 2, 3	Main protease	icw. Z1220452176	dimer	
5r80	[11]	1st	1, 2, 3	Main protease	icw. Z18197050	dimer	
5r81	[11]	2nd	1, 2, 3	Main protease	icw. Z1367324110	dimer	
5r82	[11]	2nd	1, 2, 3	Main protease	icw. Z219104216	dimer	
5r83	[11]	1st	1, 2, 3	Main protease	icw. Z44592329	dimer	
5r84	[11]	1st	1, 2, 3, 2.5	Main protease	icw. Z31792168	dimer	
5r8t	[11]	1st	1, 2, 3	Main protease		dimer	
5re4	[11]	1st	1, 2, 3, 1.1	Main protease	icw. Z1129283193	dimer	
5re5	[11]	1st	1, 2, 3	Main protease	icw. Z1129283193	dimer	
5re6	[11]	1st	1, 2, 3	Main protease	icw. Z1129283193	dimer	
5re7	[11]		1, 2, 3	Main protease	icw. Z30932204	dimer	bond distance between intraresidue atoms 945 and 950 exceeds 6 Å
5re8	[11]	1st	1, 2, 3	Main protease	icw. Z2737076969	dimer	
5re9	[11]		1, 2, 3	Main protease	icw. Z2856434836	dimer	bond distance between intraresidue atoms 945 and 950 exceeds 6 Å
5rea	[11]		1, 2, 3	Main protease	icw. Z31432226	dimer	bond distance between intraresidue atoms 954 and 979 exceeds 6 Å
5reb	[11]	1st	1, 2, 3	Main protease	icw. Z2856434899	dimer	
5rec	[11]	1st	1, 2, 3, 0.4	Main protease	icw. Z1587220559	dimer	
5red	[11]		1, 2, 3	Main protease	icw. Z2856434865	dimer	bond distance between intraresidue atoms 952 and 957 exceeds 6 Å
5ree	[11]	1st	1, 2, 3	Main protease	icw. Z2217052426	dimer	
5ref	[11]	1st	1, 2, 3	Main protease	icw. Z24758179	dimer	
5reg	[11]		1, 2, 3	Main protease	icw. Z1545313172	dimer	bond distance between intraresidue atoms 947 and 972 exceeds 6 Å
5reh	[11]	1st	1, 2, 3, 1.8	Main protease	icw. Z111507846	dimer	
5rei	[11]	1st	1, 2, 3	Main protease	icw. Z2856434856	dimer	
5rej	[11]	1st	1, 2, 3	Main protease	icw. PCM-0102241	dimer	

continued on next page

PDB code	Ref.	rigidity class.	E_{cut}'s (kcal/mol)	protein type	role	oligomeric state	error
5rek	[11]	1st	1, 2, 3	Main protease	icw. PCM-0102327	dimer	
5rel	[11]	2nd	1, 2, 3, 1.5	Main protease	icw. PCM-0102340	dimer	
5rem	[11]	1st	1, 2, 3	Main protease	icw. PCM-0103016	dimer	
5ren	[11]	1st	1, 2, 3	Main protease	icw. PCM-0102425	dimer	
5reo	[11]	1st	1, 2, 3, 1.7	Main protease	icw. PCM-0102578	dimer	
5rep	[11]	1st	1, 2, 3	Main protease	icw. PCM-0102201	dimer	
5rer	[11]	1st	1, 2, 3	Main protease	icw. PCM-0102615	dimer	
5res	[11]	1st	1, 2, 3	Main protease	icw. PCM-0102281	dimer	
5ret	[11]	1st	1, 2, 3	Main protease	icw. PCM-0102269	dimer	
5reu	[11]	1st	1, 2, 3	Main protease	icw. PCM-0102395	dimer	
5rev	[11]	1st	1, 2, 3	Main protease	icw. PCM-0103072	dimer	
5rew	[11]	1st	1, 2, 3	Main protease	icw. PCM-0102275	dimer	
5rex	[11]	1st	1, 2, 3	Main protease	icw. PCM-0102287	dimer	
5rey	[11]	1st	1, 2, 3, 0.5	Main protease	icw. PCM-0102911	dimer	
5rez	[11]	1st	1, 2, 3	Main protease	icw. POB0129	dimer	
5rf0	[11]	1st	1, 2, 3	Main protease	icw. POB0073	dimer	
5rf1	[11]	1st	1, 2, 3	Main protease	icw. NCL-00023830	dimer	
5rf2	[11]	1st	1, 2, 3	Main protease	icw. Z1741969146	dimer	
5rf3	[11]	1st	1, 2, 3	Main protease	icw. Z1741970824	dimer	
5rf4	[11]	1st	1, 2, 3	Main protease	icw. Z1741982125	dimer	
5rf5	[11]	1st	1, 2, 3, 1.5	Main protease	icw. Z3241250482	dimer	
5rf6	[11]	2nd	1, 2, 3	Main protease	icw. Z1348371854	dimer	
5rf7	[11]	1st	1, 2, 3	Main protease	icw. Z316425948_minor	dimer	
5rf8	[11]	1st	1, 2, 3	Main protease	icw. Z271004858	dimer	
5rf9	[11]	1st	1, 2, 3	Main protease	icw. Z217038356	dimer	
5rfa	[11]	1st	1, 2, 3	Main protease	icw. Z2643472210	dimer	
5rfb	[11]	1st	1, 2, 3	Main protease	icw. Z1271660837	dimer	
5rfc	[11]	1st	1, 2, 3	Main protease	icw. Z979145504	dimer	
5rfd	[11]	1st	1, 2, 3	Main protease	icw. Z126932614	dimer	
5rfe	[11]	1st	1, 2, 3	Main protease	icw. Z126932614	dimer	
5rff	[11]	1st	1, 2, 3	Main protease	icw. PCM-0102704	dimer	
5rfg	[11]	1st	1, 2, 3	Main protease	icw. PCM-0102372	dimer	
5rfh	[11]	1st	1, 2, 3	Main protease	icw. PCM-0102277	dimer	
5rfi	[11]	1st	1, 2, 3, 1.5	Main protease	icw. PCM-0102353	dimer	
5rfj	[11]	1st	1, 2, 3, 1.7	Main protease	icw. PCM-0103067	dimer	

continued on next page

PDB code	Ref.	rigidity class.	E_{cut}'s (kcal/mol)	protein type	role	oligomeric state	error
5rfk	11	1st	1, 2, 3	Main protease	icw. PCM-0102575	dimer	
5rfl	11	1st	1, 2, 3	Main protease	icw. PCM-0102389	dimer	
5rfm	11	1st	1, 2, 3, 0.5	Main protease	icw. PCM-0102539	dimer	
5rfn	11	1st	1, 2, 3, 1.9	Main protease	icw. PCM-0102868	dimer	
5rfo	11	1st	1, 2, 3	Main protease	n complex with PCM-0102972	dimer	
5rfp	11	2nd	1, 2, 3	Main protease	icw. PCM-0102190	dimer	
5rfq	11	1st	1, 2, 3, 1.8	Main protease	icw. PCM-0102179	dimer	
5rfr	11	1st	1, 2, 3	Main protease	icw. PCM-0102169	dimer	
5rfs	11	1st	1, 2, 3	Main protease	icw. PCM-0102739	dimer	
5rft	11	1st	1, 2, 3	Main protease	icw. PCM-0102432	dimer	
5rfu	11	1st	1, 2, 3	Main protease	icw. PCM-0102121	dimer	
5rfv	11	1st	1, 2, 3	Main protease	icw. PCM-0102306	dimer	
5rfw	11	1st	1, 2, 3, 1.5	Main protease	icw. PCM-0102243	dimer	
5rfx	11	1st	1, 2, 3	Main protease	icw. PCM-0102254	dimer	
5rfy	11	1st	1, 2, 3, 0.5 1.5	Main protease	icw. PCM-0102974	dimer	
5rfz	11	1st	1, 2, 3	Main protease	icw. PCM-0102274	dimer	
5rg0	11	1st	1, 2, 3	Main protease	icw. PCM-0102535	dimer	
5rg1	11	1st	1, 2, 3, 1.5	Main protease	icw. NCL-00024905	dimer	
5rg2	11	2nd	1, 2, 3, 2.5	Main protease	icw. NCL-00025058	dimer	
5rg3	11	1st	1, 2, 3	Main protease	icw. NCL-00025412	dimer	
5rgg	11	1st	1, 2, 3, 1.5	Main protease	icw. Z2856434890 (Mpro-x0165)	dimer	
5rgh	11	1st	1, 2, 3	Main protease	icw. Z1619978933 (Mpro-x0395)	dimer	
5rgi	11	1st	1, 2, 3	Main protease	icw. Z369936976 (Mpro-x0397)	dimer	
5rgj	11	2nd	1, 2, 3, 1.5	Main protease	icw. Z1401276297 (Mpro-x0425)	dimer	
5rgk	11	1st	1, 2, 3	Main protease	icw. Z1310876699 (Mpro-x0426	dimer	
5rgl	11	1st	1, 2, 3, 1.6	Main protease	icw. PCM-0102962 (Mpro-x0705)	dimer	
5rgm	11	1st	1, 2, 3	Main protease	icw. PCM-0102142 (Mpro-x0708)	dimer	

continued on next page

PDB code	Ref.	rigidity class.	E_{cut}'s (kcal/mol)	protein type	role	oligomeric state	error
5rgn	[11]	1st	1, 2, 3, 1.5	Main protease	icw. PCM-0102759 (Mpro-x0731)	dimer	
5rgo	[11]	1st	1, 2, 3	Main protease	icw. PCM-0102248 (Mpro-x0736)	dimer	
5rgp	[11]	1st	1, 2, 3	Main protease	icw. PCM-0102628 (Mpro-x0771)	dimer	
5rgq	[11]	1st	1, 2, 3, 1.75	Main protease	icw. Z1849009686 (Mpro-x1086)	dimer	
5rgr	[11]	1st	1, 2, 3	Main protease	icw. Z328695024 (Mpro-x1101)	dimer	
5rgs	[11]	1st	1, 2, 3	Main protease	icw. Z1259086950 (Mpro-x1163)	dimer	
6lu7	[12]	1st	1, 2, 3, 0.7 1.5	Main protease	icw. an inhibitor N3	dimer	
6lvn	[13]	brick	1, 2, 3	Spike protein HR2 domain	Spike protein does binding to receptor and involved in fusion of membranes	tetramer	
6lxt	[14]	1st	1, 2, 3, 3.3 3.6 4.6	Spike protein post fusion core	S2 subunit	trimer	
6lzg	[15]	1st	1, 2, 3	Spike protein domain icw. ace2		dimer	
6m03	[16]	1st	1, 2, 3	main protease	apoform	dimer	
6m0j	[17]		1, 2, 3	Spike protein domain icw. ace2		hetero-dimer	bond distance between intraresidue atoms 3327 and 3342 exceeds 6 Å
6m17	[18]	domain	1, 2, 3, 0.1 0.2 0.4 0.5 0.7	Ace2 icw. amino acid transporte and spike protein domain		hetero-hexamer	
6m18	[18]	domain	1, 2, 3, 0.03 0.06 0.2 0.5	ace2 icw. amino acid transporter	This structure contains no viral protein	hetero-tetramer	
6m1d	[18]	2nd	1, 2, 3, 0.2 0.4 0.6	ace2 icw. amino acid transporter	This structure contains no viral protein	hetero-tetramer	
6m2n	[19]	2nd	1, 2, 3, 0.5	Protease	structure with ligand	dimer	
6m2q	[20]	2nd	1, 2, 3	Protease	apo protein	dimer	

continued on next page

PDB code	Ref.	rigidity class.	E_{cut}'s (kcal/mol)	protein type	role	oligomeric state	error
6m3m	21	2nd	1, 2, 3, 0.15	Nucleocapside protein RNA binding domain		monomer	
6m71	22	2nd	1, 2, 3, 0.08 0.17 0.4 0.7	RNA polymerase	icw. cofactors	hetero-tetramer	
6vsb	23	domain	1, 2, 3, 0.005 0.04 0.1 0.35 0.8 1.5	Spike ecto domain	EM structure 3.5 angstrom	trimer	
6vw1	24	1st	1, 2, 3, 0.5 4.0 4.5	Spike receptor binding domain with Ace2		hetero-dimer	
6vww	25		1, 2, 3	NSP15 endoribonuclease		hexamer	bond distance between intraresidue atoms 26 and 27 exceeds 6 Å
6vxs	26	domain	1, 2, 3, 1.3	ADP ribose phosphotase		monomer	
6vxx	27	domain	1, 2, 3, 0.001 0.08 0.5 1.5	Spike ecto domain	EM structure 2.8 angstrom closed	trimer	
6vyb	27	domain	1, 2, 3, 0.001 0.5	Spike ecto domain	Em structure open structure 3.2 angstrom	trimer	
6vyo	26	brick	1, 2, 3, 0.1 0.7	nucleocapside phosphoprotein		tetramer	
6w01	25		1, 2, 3	NSP15 endoribonuclease	in the Complex with a Citrate	hexamer	bond distance between intraresidue atoms 9 and 24 exceeds 6 Å
6w02	26		1, 2, 3	ADP ribose phosphotase	complex with ADP ribose	monomer	bond distance between intraresidue atoms 2095 and 2100 exceeds 6 Å
6w41	28		1, 2, 3	Spike receptor binding domain complexed with antibody	icw. human antibody CR3022	hetero-trimer	steric clashes
6w4b	26	1st	1, 2, 3, 0.1 0.4	NSP9 RNA binding protein		dimer	

continued on next page

PDB code	Ref.	rigidity class.	E_{cut}'s (kcal/mol)	protein type	role	oligomeric state	error
6w4h	[26]		1, 2, 3	NSP16 - NSP10 Complex		hetero-dimer	bond distance between intraresidue atoms 430 and 453 exceeds 6 Å
6w61	[26]		1, 2, 3	NSP10 and NSP16 complex: methyltransferase stimulatory complex		hetero-dimer	bond distance between intraresidue atoms 320 and 345 exceeds 6 Å
6w63	[26]	2nd	1, 2, 3, 1.5	Main protease	bound to potent broad-spectrum non-covalent inhibitor X77	dimer	
6w6y	[26]	2nd	1, 2, 3	ADP ribose phosphotase	icw. AMP	monomer	
6w75	[26]		1, 2, 3	NSP10-NSP16 complex		hetero-dimer	bond distance between intraresidue atoms 322 and 323 exceeds 6 Å
6w9c	[26]	1st	1, 2, 3, 0.5	papain like protease		trimer	
6w9q	[29]	1st	1, 2, 3	NSP9 RNA replicase		dimer	
6wcf	[26]		1, 2, 3	ADP ribose phosphotase	icw. MES	monomer	bond distance between intraresidue atoms 1189 and 1214 exceeds 6 Å
6wen	[26]		1, 2, 3	ADP ribose phosphotase	apo form	monomer	bond distance between intraresidue atoms 1141 and 1147 exceeds 6 Å
6y2e	[16]	2nd	1, 2, 3	Main protease		dimer	
6y2f	[16]	1st	1, 2, 3	Main protease	monoclinic form	dimer	
6y2g	[16]	2nd	1, 2, 3, 0.5 1.5	Main protease	orthorhombic form	dimer	
6y84	[30]		1, 2, 3	Main protease	unliganded active site	dimer	bond distance between intraresidue atoms 4600 and 4603 exceeds 6 Å
6yb7	[30]	2nd	1, 2, 3, 2.5 4.0	Main protease	supercedes 6y84	dimer	

continued on next page

PDB code	Ref.	rigidity class.	E_{cut}'s (kcal/mol)	protein type	role	oligomeric state	error
6yi3	31		1, 2, 3	nucleocapside phosphoprotein	NMR structure		bond distance between intraresidue atoms 4600 and 4603 exceeds 6 Å
6yla	32	2nd	1, 2, 3, 0.7 1.5	spike receptor binding domain with Fab fragment		hetero-trimer	
7btf	22	2nd	1, 2, 3, 0.1 0.3 0.4 0.8	RNA polymerase	icw. cofactors in reduced condition	hetero-tetramer	
The following PDB files were downloaded 19 May 2020							
3r24	21	1st	1, 2, 3	transferase	RNA maturation	hetero-dimer	
6lze	16	2nd	1, 2, 3, 1.7		icw. an inhibitor 11a	dimer	
6m0k	16	2nd	1, 2, 3	main protease	icw. an inhibitor 11b	dimer	
6w37	26	brick	1, 2, 3			monomer	
6w7y	21	domain	1, 2, 3, 0.015 0.15 0.4 0.6		SARS-CoV-2 reactive human antibody CR3022	dimer	
6wey	33	1, 2, 3, 2.5 4.0 5.0				monomer	
6wiq	26	"3,2"	1, 2, 3, 0.001 0.03 0.5 1.5			hetero-tetramer	
6wji	26	domain	1, 2, 3, 0.001 0.13 0.6 1.7		Crystal Structure of C-terminal Dimerization Domain of Nucleocapsid Phosphoprotein from SARS-CoV-2	dimer	
6wjt	26		1, 2, 3			hetero-dimer	bond distance between intraresidue atoms 01952 and 01967 exceeds 6 Å
6wkp	26	brick	1, 2, 3, 0.08 0.5		monoclinic crystal form	tetramer	
6wkq	26	2nd	1, 2, 3		icw. Sinefungin	hetero-dimer	

continued on next page

PDB code	Ref.	rigidity class.	E_{cut}'s (kcal/mol)	protein type	role	oligomeric state	error
6wks-bundle1	[34]	2nd	1, 2, 3, 0.01 0.2 0.5 1.5			hetero-tetramer	
6wlc	[26]		1, 2, 3		in the Complex with Uridine-5'-Monophosphate	hexamer	bond distance between intraresidue atoms 1 and 4 exceeds 6 Å
6wnp	[26]		1, 2, 3		bound to Boceprevir at 1.45 A	dimer	bond distance between intraresidue atoms 04378 and 04381 exceeds 6 Å
6woj	[26]	2nd	1, 2, 3, 4		icw. ADP-ribose	monomer	
6wq3	[26]		1, 2, 3		icw. 7-methyl-GpppA and S-adenosyl-L-homocysteine.	hetero-dimer	bond distance between intraresidue atoms 03495 and 03516 exceeds 6 Å
6wqd	[26]		1, 2, 3		Complex of NSP7 and the C-terminal Domain of NSP8 from SARS-CoV-2	hetero-tetramer	bond distance between intraresidue atoms 359 and 376 exceeds 6 Å
6wqf	[35]		1, 2, 3		Revealed by Room Temperature X-ray Crystallography	dimer	bond distance between intraresidue atoms 03306 and 03331 exceeds 6 Å
6wrh	[26]		1, 2, 3			monomer	bond distance between intraresidue atoms 02089 and 02110 exceeds 6 Å
6wrz	[26]		1, 2, 3			hetero-dimer	bond distance between intraresidue atoms 03491 and 03510 exceeds 6 Å
6wtc	[26]	domain	1, 2, 3, 4		Second Form of the Co-factor Complex of NSP7 and the C-terminal Domain of NSP8 from SARS CoV-2	hetero-tetramer	

continued on next page

PDB code	Ref.	rigidity class.	E_{cut}'s (kcal/mol)	protein type	role	oligomeric state	error
6wvn	[26]	2nd	1, 2, 3		icw. 7-methyl-GpppA and S-Adenosylmethionine.	hetero-dimer	
6y7m-bundle1	[16]	2nd	1, 2, 3, 0.3 0.4			dimer	
6yhu	[36]	domain	1, 2, 3	nsp7-nsp8 complex of SARS-CoV-2		hetero-dimer	
6ym0	[32]		1, 2, 3		icw. CR3022 Fab	hetero-trimer	steric clashes
6ynq	[37]		1, 2, 3	main protease	bound to 2-Methyl-1-tetralone	dimer	bond distance between intraresidue atoms 062 and 085 exceeds 6 Å
6yor	[38]	2nd	1, 2, 3, 0.26	SARS-CoV-2 spike S1 protein	icw. CR3022 Fab	hetero-trimer	
6yt8	[39]		1, 2, 3	main protease	bound to pyrithione zinc	dimer	bond distance between intraresidue atoms 079 and 082 exceeds 6 Å
6yva	[40]	1st	1, 2, 3, 0.15 0.3 1.5			hetero-dimer	
6ywk	[41]	domain	1, 2, 3, 1.5	SARS-CoV-2 (Covid-19) NSP3 macrodomain	icw. HEPES	monomer	
6ywl	[41]	domain	1, 2, 3, 1.5	SARS-CoV-2 (Covid-19) NSP3 macrodomain	icw. ADP-ribose	monomer	
6ywm	[41]	domain	1, 2, 3, 2.5 4.0	SARS-CoV-2 (Covid-19) NSP3 macrodomain	icw. MES	monomer	
6yyt	[42]	2nd	1, 2, 3, 0.004 0.15 0.3 0.4	replicating SARS-CoV-2 polymerase		hetero-tetramer	
6yz1	[43]		1, 2, 3		complex with Sinefungin	hetero-dimer	bond distance between intraresidue atoms 03770 and 03773 exceeds 6 Å

continued on next page

PDB code	Ref.	rigidity class.	E_{cut}'s (kcal/mol)	protein type	role	oligomeric state	error
7bqy	[12]	2nd	1, 2, 3, 0.5 1.2	main protease	icw. AN INHIBITOR N3 at 1.7 angstrom	dimer	
7bro	[44]	2nd	1, 2, 3, 1.3 1.5	main protease		dimer	
7brp	[44]	2nd	1, 2, 3, 0.5 0.75 1.3	main protease	complexed with Boceprevir	dimer	
7brr	[44]	1st	1, 2, 3, 1.5	main protease	complexed with GC376	dimer	
7buy	[12]	2nd	1, 2, 3, 0.5	main protease	icw. carmofur	dimer	
7bv1	[45]	2nd	1, 2, 3, 0.05 0.15 0.25 0.5 1.5	nsp12-nsp7-nsp8 complex	apo?	hetero-tetramer	
7bv2	[45]	2nd	1, 2, 3, 0.04 0.15 0.4	nsp12-nsp7-nsp8 complex	bound to the template-primer RNA and triphosphate form of Remdesivir(RTP)	hetero-trimer	
7bz5	[46]	brick	1, 2, 3	COVID-19 virus spike receptor-binding domain	complexed with a neutralizing antibody	hetero-trimer	
The following PDB files were downloaded 29 May 2020							
5rgt	[11]		1, 2, 3	main protease	icw. Z4439011607	dimer	bond distance between intraresidue atoms 03302 and 03325 exceeds 6 Å
5rgu	[11]	1st	1, 2, 3, 2.5	main protease	icw. Z4444622180	dimer	
5rgv	[11]	1st	1, 2, 3	main protease	icw. Z4444622066	dimer	
5rgw	[11]	1st	1, 2, 3	main protease	icw. Z4444621910	dimer	
5rgx	[11]	1st	1, 2, 3	main protease	icw. Z1344037997	dimer	
5rgy	[11]	brick	1, 2, 3, 1.7 1.9	main protease	icw. Z1535580916	dimer	
5rgz	[11]	1st	1, 2, 3	main protease	icw. Z1343543528	dimer	
5rh0	[11]	1st	1, 2, 3	main protease	icw. Z1286870272	dimer	
5rh1	[11]	1st	1, 2, 3, 1.7	main protease	icw. Z2010253653	dimer	
5rh2	[11]	2nd	1, 2, 3	main protease	icw. Z1129289650	dimer	

continued on next page

PDB code	Ref.	rigidity class.	E_{cut}'s (kcal/mol)	protein type	role	oligomeric state	error
5rh3	[11]		1, 2, 3	main protease	icw. Z1264525706	dimer	bond distance between intraresidue atoms 03302 and 03325 exceeds 6 Å
5rh4	[11]		1, 2, 3	main protease	icw. Z1530425063	dimer	bond distance between intraresidue atoms 02536 and 02551 exceeds 6 Å
5rh5	[11]	1st	1, 2, 3, 2.1 2.4	main protease	icw. Z4439011520	dimer	
5rh6	[11]	2nd	1, 2, 3	main protease	icw. Z4439011588	dimer	
5rh7	[11]		1, 2, 3	main protease	icw. Z4439011584	dimer	bond distance between intraresidue atoms 03310 and 03333 exceeds 6 Å
5rh8	[11]	1st	1, 2, 3, 1.5	main protease	icw. Z4444621965	dimer	
5rh9	[11]		1, 2, 3	main protease	icw. Z4438424255	dimer	bond distance between intraresidue atoms 03302 and 03325 exceeds 6 Å
5rha	[11]		1, 2, 3	main protease	icw. Z147647874	dimer	bond distance between intraresidue atoms 03302 and 03327 exceeds 6 Å
6wps	[47]	domain	1, 2, 3	SARS-CoV-2 spike glycoprotein	icw. the S309 neutralizing antibody Fab fragment	Hetero-9-mer	
6wpt	[47]	domain	1, 2, 3	SARS-CoV-2 spike glycoprotein	icw. the S309 neutralizing antibody Fab fragment (open state)	Hetero-7-mer	
6wtj	[48]	2nd	1, 2, 3	main protease	Feline coronavirus drug inhibits	dimer	
6wtk	[48]	1st	1, 2, 3	main protease	Feline coronavirus drug inhibits	dimer	
6wtm	[48]	2nd	1, 2, 3	main protease	Feline coronavirus drug inhibits	dimer	
6wtt	[49]	2nd	1, 2, 3	main protease	with inhibitor GC-376	dimer	

continued on next page

PDB code	Ref.	rigidity class.	E_{cut}'s (kcal/mol)	protein type	role	oligomeric state	error
6wuu	50	1st	1, 2, 3, 0.5	Papain-like protease	icw. peptide inhibitor VIR250	hetero-dimer	
6wx4	50		1, 2, 3	Papain-like protease	icw. peptide inhibitor VIR251	monomer	steric clashes
6wxc	26		1, 2, 3	NSP15 Endoribonuclease	n the Complex with potential repurposing drug Tipiracil	hexamer	bond distance between intraresidue atoms 1 and 4 exceeds 6 Å
6wxd	51	brick	1, 2, 3	Nsp9 RNA-replicase		dimer	
6wzo	52	domain	1, 2, 3, 0.5	"Nucleocapsid dimerization domain, P1 form"		dimer	
6wzq	52	domain	1, 2, 3	"Nucleocapsid dimerization domain, P21 form"		dimer	
6wzu	26		1, 2, 3	Papain-Like Protease	P3221 space group	monomer	bond distance between intraresidue atoms 02023 and 02026 exceeds 6 Å
6x1b	26		1, 2, 3	NSP15 Endoribonuclease	in the Complex with the Product Nucleotide GpU.	hexamer	bond distance between intraresidue atoms 05377 and 05380 exceeds 6 Å
6x29	53	domain	1, 2, 3, 0.002 0.05 0.3			trimer	
6x2a	53	domain	1, 2, 3, 0.005 0.1			trimer	
6x2b	53	domain	1, 2, 3, 0.005 0.14 0.5			trimer	
6x2c	53	domain	1, 2, 3, 0.001 0.05 0.25 0.7			trimer	
6yun	54	domain	1, 2, 3	C-terminal Dimerization Domain of Nucleocapsid Phosphoprotein		dimer	bond distance between intraresidue atoms 03404 and 03429 exceeds 6 Å

continued on next page

PDB code	Ref.	rigidity class.	E_{cut}'s (kcal/mol)	protein type	role	oligomeric state	error
6yvf	[37]		1, 2, 3	Main Protease	bound to AZD6482	dimer	steric clashes
6yz6	[37]	1st	1, 2, 3		hemiacetal complex of Main Protease and Leupeptin	dimer	
7bw4	[55]	2nd	1, 2, 3, 0.015 0.15	RNA-dependent RNA polymerase		hetero-tetramer	
7c01	[56]	2nd	1, 2, 3		potent human neutralizing antibody targeting SARS-CoV-2 RBD	hetero-trimer	
7c22	[57]	domain	1, 2, 3, 0.5	C-terminal domain of SARS-CoV-2 nucleocapsid protein		dimer	
7c2i	[58]	2nd	1, 2, 3	nsp16-nsp10 heterodimer	icw. SAM (with additional SAM during crystallization)	hetero-dimer	
7c2j	[58]	2nd	1, 2, 3, 1.3	nsp16-nsp10 heterodimer	icw. SAM (with additional SAM during crystallization)	hetero-dimer	
The following PDB files were downloaded 27 July 2020							
5rhb	[11]	1st	1, 2, 3	main protease		dimer	
5rhc	[11]	1st	1, 2, 3	main protease		dimer	
5rhd	[11]	1st	1, 2, 3, 3.2	main protease		dimer	
5rhe	[11]	1st	1, 2, 3	main protease		dimer	
5rhf	[11]	1st	1, 2, 3	main protease		dimer	
6m1v	[59]	domain, brick	1, 2, 3, 4.6, 5.05	post fusion core of S2 of spike protein	part of the spike protein	trimer	
6m5i	[60]	domain	1, 2, 3, 0.1	nsp7-nsp8c complex	potentially interesting as involved in RNA replication	dimer	
6x4i	[26]		1, 2, 3	endoribonuclease		6-mer	bond distance between intraresidue atoms 00003 and 00018 exceeds 6 Å
6x6p	[61]	domain	1, 2, 3, 0.01, 0.15, 0.25, 0.5	spike	another spike structure, determined via EM at 3.2 Å resolution	trimer	
6xa4	[62]	2nd	1, 2, 3, 1.5	main protease		dimer	

continued on next page

PDB code	Ref.	rigidity class.	E_{cut}'s (kcal/mol)	protein type	role	oligomeric state	error
6xa9	[63]		1, 2, 3	another protease (not the main one)		hetero-dimer	bond distance between intraresidue atoms 04000 and 04003 exceeds 6 Å
6xaa	[64]		1, 2, 3	same protease as 6xa9 but different complex		hetero-dimer	bond distance between intraresidue atoms 01520 and 01523 exceeds 6 Å
6xb0	[65]		1, 2, 3	main protease		dimer	bond distance between intraresidue atoms 03354 and 03381 exceeds 6 Å
6xb1	[66]	1st	1, 2, 3	main protease		dimer	
6xb2	[67]	2nd	1, 2, 3, 0.5	main protease		dimer	
6xbg	[68]	2nd	1, 2, 3	main protease		dimer	
6xbh	[69]		1, 2, 3	main protease		dimer	bond distance between intraresidue atoms 04558 and 04563 exceeds 6 Å
6xbi	[70]	2nd	1, 2, 3	main protease		dimer	
6xc2	[71]	2nd, brick	1, 2, 3	part of the spike protein		hetero-trimer	
6xc3	[71]	brick	1, 2, 3, 0.5	part of the spike protein		hetero-5-mer	
6xc4	[71]	3 0	1, 2, 3	part of the spike protein		hetero-trimer	
6xc7	[71]	2nd	1, 2, 3, 0.5, 1.5	part of the spike protein		hetero-5-mer	
6xch	[72]	1 2?	1, 2, 3, 0.5, 1.5	main protease		dimer	
6xcm	[73]	domain	1, 2, 3, 0.005, 0.05, 0.2, 0.5	spike protein		hetero-7-mer	

continued on next page

PDB code	Ref.	rigidity class.	E_{cut}'s (kcal/mol)	protein type	role	oligomeric state	error
6xcn	73	domain	1, 2, 3, 0.001, 0.02, 0.08, 0.5	spike protein		hetero-9-mer	"missing residues, steric clashes"
6xdc	74	domain	1, 2, 3, 0.08, 0.25, 0.5	protein from open reading frame 3	protein may be part important in the inflamatory response which is critical to understanding why some patients get very ill and others not https://faseb.onlinelibrary.wiley.com/doi/abs/10.1096/fj.201802418R	dimer	
6xdg	75	domain	1, 2, 3, 0.008, 0.04, 0.2	spike protein		hetero-5-mer	
6xdh	47	1st	1, 2, 3, 0.5, 0.75	another endoribonuclease		trimer	
6xe1	76		1, 2, 3		part of the spike protein	hetero-trimer	no chain A
6xfn	77	2nd	1, 2, 3, 0.75	main protease		dimer	
6xg3	26		1, 2, 3	This is a protease but not the main one		monomer	bond distance between intraresidue atoms 02024 and 02045 exceeds 6 Å
6xhm	78	1st, 2nd	1, 2, 3, 1.5	main protease		dimer	
6xhu	79	1st, 2nd	1, 2, 3, 0.5	main protease		dimer	
6xip	26	2nd	1, 2, 3, 3.5, 4.5	nsp7 and 8	similar to 6m5i structure but at low resolution	hetero-tetramer	
6xkf	80	1st, 2nd	1, 2, 3, 0.25	nsp7 and 9	similar to 6m5i structure but at low resolution	homo-dimer	
6xkh	81	2nd	1, 2, 3, 3.5	main protease		dimer	
6xkl	82	domain	1, 2, 3, 0.05, 0.15, 0.5	spike protein		trimer	

continued on next page

PDB code	Ref.	rigidity class.	E_{cut}'s (kcal/mol)	protein type	role	oligomeric state	error
6xkm	[26]	2nd	1, 2, 3, 0.25, 1.5	nsp10/nsp16 methyl transferase		hetero-dimer	
6xmk	[83]	2nd	1, 2, 3	main protease	low resolution structure	dimer	
6xoa	[84]	1st, 2nd	1, 2, 3, 0.2	main protease		dimer	
6yz5	[85]		1, 2, 3	part of the spike protein		hetero-dimer	no chain A
6z2m	[86]	2 0	1, 2, 3, 0.4, 1.5	part of the spike protein		hetero-tetramer	
6z43	[87]	domain	1, 2, 3, 0.05, 0.25	spike protein		hetero-6-mer	
6z4u	[88]	domain	1, 2, 3, 0.001, 0.5	Orf9b	protein may be involved in immune regulation which is important in understanding the different responses of different patients to infection	homo-dimer	
6z97	[32]	domain	1, 2, 3, 0.001, 0.01, 0.07, 0.25, 0.5	spike protein		trimer	
6zco	[89]		1, 2, 3	spike protein		dimer	bond distance between intraresidue atoms 00202 and 00225 exceeds 6 Å
6zct	[90]	1 0	1, 2, 3, 0.5	nsp10	forms part of the methyl transferase complex	monomer	
6zcz	[91]		1, 2, 3	part of the spike protein		hetero-tetramer	no chain A
6zdh	[91]	domain	1, 2, 3, 0.002, 0.05, 0.2, 0.5	spike protein		hetero-9-mer	

continued on next page

PDB code	Ref.	rigidity class.	E_{cut}'s (kcal/mol)	protein type	role	oligomeric state	error
6zer	[91]	1st, 2nd	1, 2, 3, 0.05, 0.25, 0.75	part of the spike protein		hetero-trimer	
6zfo	[91]	2nd	1, 2, 3, 0.1, 0.5	part of the spike protein		hetero-trimer	
6zge	[92]		1, 2, 3	spike protein		trimer	bond distance between intraresidue atoms 12073 and 12092 exceeds 6 Å
6zgg	[93]	domain	1, 2, 3, 0.002, 0.02, 0.03, 0.1, 0.25	spike protein		trimer	
6zgh	[94]	domain	1, 2, 3, 0.001, 0.03, 0.075, 0.2, 4.2	spike protein		trimer	
6zgi	[95]	2nd	1, 2, 3	spike protein		trimer	bond distance between intraresidue atoms 12073 and 12092 exceeds 6 Å
7bq7	[96]	domain	1, 2, 3, 0.25	nsp10/nsp16 methyl transferase		hetero-dimer	
7bwj	[97]		1, 2, 3	part of the spike protein		hetero-trimer	no chain A
7byr	[98]	domain	1, 2, 3, 0.001, 0.03, 0.07, 0.15, 0.5	spike protein		hetero-5-mer	
7bzf	[15]	2nd, domain	1, 2, 3, 0.02, 0.1, 0.25, 0.5	RNA polymerase	important for replication	hetero-tetramer	
7c2k	[15]	1st, 2nd	1, 2, 3, 0.15, 0.25, 0.4	Complex of proteins needed in replication	chain A is the RNA polymerase which is the important one	hetero-tetramer	

continued on next page

PDB code	Ref.	rigidity class.	E_{cut}'s (kcal/mol)	protein type	role	oligomeric state	error
7c2l	[99]	domain	1, 2, 3, 0.003, 0.08, 0.15, 0.5	spike protein		hetero-9-mer	
7c8r	[100]	1st, 2nd	1, 2, 3	main protease		dimer	
7c8t	[101]	1st, 2nd	1, 2, 3	main protease		dimer	
7c8u	[102]	1st, 2nd	1, 2, 3, 0.5	main protease		dimer	
7c8v	[103]	brick	1, 2, 3	part of the spike protein		hetero-dimer	
7c8w	[104]		1, 2, 3	art of the spike protein		hetero-dimer	bond distance between intraresidue atoms 00965 and 00966 exceeds 6 Å
7can	[105]		1, 2, 3	part of the spike protein		hetero-dimer	bond distance between intraresidue atoms 00966 and 00989 exceeds 6 Å

Table S1. List of all SARS-Cov-2-related structures investigated in this study. The relevant references have been provided if they had been listed on the PDB download pages for each PDB code at the time of download access. The abbreviation "icw." stands for "in complex with". Two large scale collaborations have been labelled by acronyms PANDDA[11] and CSGID[26]. The rigidity association into solid "brick", 1st and 2nd order as well as rigidity domains are given on column 3. Values for E_{cut} are given explicitly whereas all results have been computed for all 6 modes m_7 to m_{12}. Protein description, role and oligomeric state are as written of from the information in each PDB entry or in the referenced publication. The last column indicates the computational error encountered when no motion result has been computed.

References

1. RA Römer, NS Römer, AK Wallis, Flex-Covid19 data repository, https://warwick.ac.uk/flex-covid19-data (2020).
2. RA Römer, NS Römer, AK Wallis, 6vxx-monomer, https://livewarwickac.sharepoint.com/:v:/s/Flex-Covid19/EQern4arlFtFmBJgcdJGZfoBRXlS4foHGWeL_CY-R8qDVA?e=JdhKHS (2020).
3. RA Römer, NS Römer, AK Wallis, 6lu7-dimer, https://livewarwickac.sharepoint.com/:v:/s/Flex-Covid19/EW5Tj2CCMfJMop2RCxJNSFYBBbiFaeNLc9MC1eNfxX-lug?e=w5883E (2020).
4. RA Römer, NS Römer, AK Wallis, 6vxx-trimer-side, https://livewarwickac.sharepoint.com/:v:/s/Flex-Covid19/EVRO00oUOFJGlnys98iQ3W0BeQPnxIH92GkwP9w_GuKzzg?e=XTLwT7 (2020).
5. RA Römer, NS Römer, AK Wallis, 6vxx-trimer-top, https://livewarwickac.sharepoint.com/:v:/s/Flex-Covid19/EXfYLrNIaJNHpR-VJjjfbysB8yqrHSRHoTfSTeEjox4_XQ?e=84I094 (2020).
6. RA Römer, NS Römer, AK Wallis, 6vyb-trimer-side, https://livewarwickac.sharepoint.com/:v:/s/Flex-Covid19/EUETBeGAatZOp4SGFew7P6ABPY1LSPsXNTm_ck2JjiGnaw?e=m4SRzh (2020).
7. RA Römer, NS Römer, AK Wallis, 6vyb-trimer-top, https://livewarwickac.sharepoint.com/:v:/s/Flex-Covid19/EShhXMwZf8NMl2FdmQBARFsBxV9DbFR7K2bJpEMfys6Njg?e=sX5sbG (2020).
8. RA Römer, NS Römer, AK Wallis, rigidity dilution decomposition plot for 6lu7, https://files.warwick.ac.uk/rroemer/files/6lu7-dimer_hydro.ps (2020).
9. RA Römer, NS Römer, AK Wallis, rigidity dilution decomposition plot for 6vxx, https://files.warwick.ac.uk/rroemer/files/6vxx-trimer_hydro.ps (2020).
10. RA Römer, NS Römer, AK Wallis, rigidity dilution decomposition plot for 6vyb, https://files.warwick.ac.uk/rroemer/files/6vyb-trimer_hydro.ps (2020).
11. PanDDA analysis group. PanDDA analysis group deposition (2020).
12. Jin, Z. *et al.* Structure of M pro from COVID-19 virus and discovery of its inhibitors. *Nature* DOI: 10.1038/s41586-020-2223-y (2020).
13. Zhu, Y. & Sun, F. RCSB PDB - 6LVN: Structure of the 2019-nCoV HR2 Domain.
14. Xia, S. *et al.* Inhibition of SARS-CoV-2 (previously 2019-nCoV) infection by a highly potent pan-coronavirus fusion inhibitor targeting its spike protein that harbors a high capacity to mediate membrane fusion. *Cell Res.* **30**, 343–355, DOI: 10.1038/s41422-020-0305-x (2020).
15. Wang, Q. Q. *et al.* Structural and Functional Basis of SARS-CoV-2 Entry by Using Human ACE2. *Cell* **181**, 894–904, DOI: 10.1016/j.cell.2020.03.045 (2020).
16. Zhang, L. *et al.* Crystal structure of SARS-CoV-2 main protease provides a basis for design of improved a-ketoamide inhibitors. Tech. Rep. (2020). DOI: 10.1126/science.abb3405.
17. Lan, J. *et al.* Structure of the SARS-CoV-2 spike receptor-binding domain bound to the ACE2 receptor. *Nature* **581**, 215–220, DOI: 10.1038/s41586-020-2180-5 (2020).
18. Yan, R. *et al.* Structural basis for the recognition of SARS-CoV-2 by full-length human ACE2. *Science* **367**, 1444–1448, DOI: 10.1126/science.abb2762 (2020).
19. Su, H., Zhao, W., Li, M., Xie, H. & Xu, Y. RCSB PDB - 6M2N: SARS-CoV-2 3CL protease (3CL pro) in complex with a novel inhibitor.
20. Su, H., Zhao, W., Li, M., Xie, H. & Xu, Y. RCSB PDB - 6M2Q: SARS-CoV-2 3CL protease (3CL pro) apo structure (space group C21).
21. Chen, Y. *et al.* Biochemical and Structural Insights into the Mechanisms of SARS Coronavirus RNA Ribose 2'-O-Methylation by nsp16/nsp10 Protein Complex. *PLoS Pathog.* **7**, e1002294, DOI: 10.1371/journal.ppat.1002294 (2011).
22. Gao, Y. *et al.* Structure of the RNA-dependent RNA polymerase from COVID-19 virus. *Science* **368**, 779–782, DOI: 10.1126/science.abb7498 (2020).
23. Wrapp, D. *et al.* Cryo-EM structure of the 2019-nCoV spike in the prefusion conformation. *Science* **367**, 1260–1263, DOI: 10.1126/science.abb2507 (2020).
24. Shang, J. *et al.* Structural basis of receptor recognition by SARS-CoV-2. *Nature* **581**, 221–224, DOI: 10.1038/s41586-020-2179-y (2020).

25. Kim, Y. *et al.* Crystal structure of Nsp15 endoribonuclease NendoU from SARS-Co-2. *Protein Sci.* pro.3873, DOI: 10.1002/pro.3873 (2020).

26. CSGID. CSGID analysis group (2020).

27. Walls, A. C. *et al.* Structure, Function, and Antigenicity of the SARS-CoV-2 Spike Glycoprotein. *Cell* **181**, 281–292, DOI: 10.1016/j.cell.2020.02.058 (2020).

28. Yuan, M. *et al.* A highly conserved cryptic epitope in the receptor binding domains of SARS-CoV-2 and SARS-CoV, DOI: 10.1126/science.abb7269 (2020).

29. Littler, D., Gully, B., Riboldi-Tunnicliffe, A. & Rossjohn, J. RCSB PDB - 6W9Q: Peptide-bound SARS-CoV-2 Nsp9 RNA-replicase.

30. Owen, C. *et al.* RCSB PDB - 6Y84: SARS-CoV-2 main protease with unliganded active site (2019-nCoV, coronavirus disease 2019, COVID-19).

31. Veverka, V. & Boura, E. RCSB PDB - 6YI3: The N-terminal RNA-binding domain of the SARS-CoV-2 nucleocapsid phosphoprotein.

32. Huo, J. *et al.* Neutralization of SARS-CoV-2 by Destruction of the Prefusion Spike. *Cell Host Microbe* DOI: 10.1016/j.chom.2020.06.010 (2020).

33. Vuksanovic, N. & Silvaggi, N. RCSB PDB - 6WEY: High-resolution structure of the SARS-CoV-2 NSP3 Macro X domain.

34. Gupta, Y., Viswanathan, T., Arya, S. & Qi, S. RCSB PDB - 6WKS: Structure of SARS-CoV-2 nsp16/nsp10 ternary complex.

35. Kneller, D., Kovalevsky, A. & Coates, L. RCSB PDB - 6WQF: Structural Plasticity of the SARS-CoV-2 3CL Mpro Active Site Cavity Revealed by Room Temperature X-ray Crystallography.

36. Konkolova, E., Klima, M. & Boura, E. RCSB PDB - 6YHU: Crystal structure of the nsp7-nsp8 complex of SARS-CoV-2.

37. Guenther, S. *et al.* RCSB PDB - 6YNQ: Structure of SARS-CoV-2 Main Protease bound to 2-Methyl-1-tetralone.

38. Huo, J. *et al.* RCSB PDB - 6YOR: Structure of the SARS-CoV-2 spike S1 protein in complex with CR3022 Fab.

39. Guenther, S. *et al.* RCSB PDB - 6YT8: Structure of SARS-CoV-2 Main Protease bound to pyrithione zinc.

40. Shin, D. & Dikic, I. RCSB PDB - 6YVA: PLpro-C111S with mISG15.

41. Ni, X. *et al.* Structural Genomics Consortium.

42. Hillen, H. *et al.* RCSB PDB - 6YYT: Structure of replicating SARS-CoV-2 polymerase.

43. Krafcikova, P., Silhan, J., Nencka, R. & Boura, E. RCSB PDB - 6YZ1: The crystal structure of SARS-CoV-2 nsp10-nsp16 methyltransferase complex with Sinefungin.

44. Fu, L. RCSB PDB - 7BRO: Crystal structure of the 2019-nCoV main protease.

45. Yin, W. *et al.* RCSB PDB - 7BV1: Cryo-EM structure of the apo nsp12-nsp7-nsp8 complex.

46. Wu, Y., Qi, J. & Gao, F. RCSB PDB - 7BZ5: Structure of COVID-19 virus spike receptor-binding domain complexed with a neutralizing antibody.

47. Pinto, D. *et al.* Seattle Structural Genomics Center for Infectious Disease.

48. Khan, M., Arutyunova, E., Young, H. & Lemieux, M. RCSB PDB - 6WTJ: Feline coronavirus drug inhibits the main protease of SARS-CoV-2 and blocks virus replication.

49. Sacco, M., Ma, C., Chen, Y. & Wang, J. RCSB PDB - 6WTT: Crystals Structure of the SARS-CoV-2 (COVID-19) main protease with inhibitor GC-376.

50. Lv, Z. & Olsen, S. RCSB PDB - 6WUU: Crystal structure of the SARS CoV-2 Papain-like protease in complex with peptide inhibitor VIR250.

51. Littler, D., Gully, B., Riboldi-Tunnicliffe, A. & Rossjohn, J. RCSB PDB - 6WXD: SARS-CoV-2 Nsp9 RNA-replicase.

52. Ye, Q. & Corbett, K. RCSB PDB - 6WZO: Structure of SARS-CoV-2 Nucleocapsid dimerization domain, P1 form.

53. Henderson, R. & Acharya, P. RCSB PDB - 6X29: SARS-CoV-2 rS2d Down State Spike Protein Trimer.

54. Zinzula, L., Basquin, J., Nagy, I. & Bracher, A. RCSB PDB - 6YUN: 1.45 Angstrom Resolution Crystal Structure of C-terminal Dimerization Domain of Nucleocapsid Phosphoprotein from SARS-CoV-2.

55. Peng, Q., Peng, R. & Shi, Y. RCSB PDB - 7BW4: Structure of the RNA-dependent RNA polymerase from SARS-CoV-2.

56. Shi, R., Qi, J., Wang, Q., Gao, F. & Yan, J. RCSB PDB - 7C01: Molecular basis for a potent human neutralizing antibody targeting SARS-CoV-2 RBD.

57. Zhou, R., Zeng, R. & Lei, J. RCSB PDB - 7C22: Crystal structure of the C-terminal domain of SARS-CoV-2 nucleocapsid protein.

58. Lin, S. *et al.* RCSB PDB - 7C2I: Crystal structure of nsp16-nsp10 heterodimer from SARS-CoV-2 in complex with SAM (with additional SAM during crystallization).

59. Sun, H., Song, H. & Wang, Q. RCSB PDB - 6M1V: Crystal structure of post fusion core of 2019-nCoV S2 subunit (2020).

60. Yan, L., Ge, J., Lou, Z. & Rao, Z. RCSB PDB - 6M5I: Crystal structure of 2019-nCoV nsp7-nsp8c complex.

61. RCSB PDB - 6X6P: Characterization of the SARS-CoV-2 S Protein: Biophysical, Biochemical, Structural, and Antigenic Analysis.

62. Sacco, M., Chen, Y. & Ma, C. RCSB PDB - 6XA4: Crystal structure of the SARS-CoV-2 (COVID-19) main protease in complex with UAW241.

63. Klemm, T. RCSB PDB - 6XA9: SARS CoV-2 PLpro in complex with ISG15 C-terminal domain propargylamide.

64. Klemm, T. RCSB PDB - 6XAA: SARS CoV-2 PLpro in complex with ubiquitin propargylamide.

65. Kneller, D. *et al.* RCSB PDB - 6XB0: Room temperature X-ray crystallography reveals catalytic cysteine in the SARS-CoV-2 3CL Mpro is highly reactive: Insights for enzyme mechanism and drug design.

66. Kneller, D. *et al.* RCSB PDB - 6XB1: Room temperature X-ray crystallography reveals catalytic cysteine in the SARS-CoV-2 3CL Mpro is highly reactive: Insights for enzyme mechanism and drug design.

67. Kneller, D. *et al.* RCSB PDB - 6XB2: Room temperature X-ray crystallography reveals catalytic cysteine in the SARS-CoV-2 3CL Mpro is highly reactive: Insights for enzyme mechanism and drug design.

68. Ma, C., Sacco, M., Chen, Y. & Wang, J. RCSB PDB - 6XBG: Crystal structure of the SARS-CoV-2 (COVID-19) main protease in complex with inhibitor UAW246.

69. Ma, C., Sacco, M., Chen, Y. & Wang, J. RCSB PDB - 6XBH: Crystal structure of the SARS-CoV-2 (COVID-19) main protease in complex with inhibitor UAW247.

70. Ma, C., Sacco, M., Chen, Y. & Wang, J. RCSB PDB - 6XBI: Crystal structure of the SARS-CoV-2 (COVID-19) main protease in complex with inhibitor UAW248.

71. Yuan, M. *et al.* RCSB PDB - 6XC2: Crystal structure of SARS-CoV-2 receptor binding domain in complex with neutralizing antibody CC12.1.

72. Kneller, D., Coates, L. & Kovalevsky, A. RCSB PDB - 6XCH: Structure of the complex between the SARS-CoV-2 Main Protease and Leupeptin.

73. Barnes, C. O. *et al.* Structures of Human Antibodies Bound to SARS-CoV-2 Spike Reveal Common Epitopes and Recurrent Features of Antibodies. *Cell* DOI: 10.1016/j.cell.2020.06.025 (2020).

74. Kern, D. M. *et al.* Cryo-EM structure of the SARS-CoV-2 3a ion channel in lipid nanodiscs. *bioRxiv : preprint server for biology* DOI: 10.1101/2020.06.17.156554 (2020).

75. Hansen, J. *et al.* Studies in humanized mice and convalescent humans yield a SARS-CoV-2 antibody cocktail. *Science* eabd0827, DOI: 10.1126/science.abd0827 (2020).

76. Hurlburt, N. K. *et al.* Structural basis for potent neutralization of SARS-CoV-2 and role of antibody affinity maturation. *bioRxiv : preprint server for biology* DOI: 10.1101/2020.06.12.148692 (2020).

77. Sacco, M., Chen, Y. & Ma, C. RCSB PDB - 6XFN: Crystal structure of the SARS-CoV-2 (COVID-19) main protease in complex with UAW243.

78. Hoffman, R. *et al.* RCSB PDB - 6XHM: Covalent complex of SARS-CoV-2 main protease with N-[(2S)-1-({(2S,3S)-3,4-dihydroxy-1-[(3S)-2-oxopyrrolidin-3-yl]butan-2-yl}amino)-4-methyl-1-oxopentan-2-yl]-4-methoxy-1H-indole-2-carboxamide.

79. Kneller, D. *et al.* RCSB PDB - 6XHU: Room temperature X-ray crystallography reveals oxidation and reactivity of cysteine residues in SARS-CoV-2 3CL Mpro: Insights for enzyme mechanism and drug design.

80. Tan, K. *et al.* RCSB PDB - 6XKF: The crystal structure of 3CL MainPro of SARS-CoV-2 with oxidized Cys145 (Sulfenic acid cysteine).

81. Tan, K. *et al.* RCSB PDB - 6XKH: THE 1.28A CRYSTAL STRUCTURE OF 3CL MAINPRO OF SARS-COV-2 WITH OXIDIZED C145 (sulfinic acid cysteine).

82. Wrapp, D., Hsieh, C.-L., Goldsmith, J. & McLellan, J. RCSB PDB - 6XKL: SARS-CoV-2 HexaPro S One RBD up.

83. Rathnayake, A. *et al.* RCSB PDB - 6XMK: 1.70 A resolution structure of SARS-CoV-2 3CL protease in complex with inhibitor 7j.

84. Tan, K., Maltseva, N., Welk, L., Jedrzejczak, R. & Joachimiak, A. RCSB PDB - 6XOA: The crystal structure of 3CL MainPro of SARS-CoV-2 with C145S mutation.

85. Naismith, J. RCSB PDB - 6YZ5: H11-D4 complex with SARS-CoV-2 RBD.

86. Naismith, J. RCSB PDB - 6Z2M: H11-D4, SARS-CoV-2 RBD, CR3022 ternary complex.

87. Huo, J. *et al.* RCSB PDB - 6Z43: Cryo-EM Structure of SARS-CoV-2 Spike : H11-D4 Nanobody Complex.

88. Weeks, S., De Graef, S. & Munawar, A. RCSB PDB - 6Z4U: X-ray Crystallographic Structure of Orf9b from SARS-CoV-2.

89. Zinzula, L., Basquin, J., Bracher, A. & Baumeister, W. RCSB PDB - 6ZCO: Crystal Structure of C-terminal Dimerization Domain of Nucleocapsid Phosphoprotein from SARS-CoV-2, crystal form II.

90. Rogstam, A. *et al.* RCSB PDB - 6ZCT: Nonstructural protein 10 (nsp10) from SARS CoV-2.

91. Zhou, D. *et al.* Structural basis for the neutralization of SARS-CoV-2 by an antibody from a convalescent patient. *Nat. Struct. Mol. Biol.* DOI: 10.1038/s41594-020-0480-y (2020).

92. Wrobel, A. *et al.* RCSB PDB - 6ZGE: Uncleavable Spike Protein of SARS-CoV-2 in Closed Conformation.

93. Wrobel, A. *et al.* RCSB PDB - 6ZGG: Furin Cleaved Spike Protein of SARS-CoV-2 with One RBD Erect.

94. Wrobel, A. *et al.* RCSB PDB - 6ZGH: Furin Cleaved Spike Protein of SARS-CoV-2 in Intermediate Conformation.

95. Wrobel, A. *et al.* RCSB PDB - 6ZGI: Furin Cleaved Spike Protein of SARS-CoV-2 in Closed Conformation.

96. Yan, L., Huang, Y., Lou, Z. & Rao, Z. RCSB PDB - 7BQ7: Crystal structure of 2019-nCoV nsp16-nsp10 complex.

97. Ju, B. *et al.* Human neutralizing antibodies elicited by SARS-CoV-2 infection. *Nature* **584**, 115–119, DOI: 10.1038/s41586-020-2380-z (2020).

98. Cao, Y. *et al.* Potent Neutralizing Antibodies against SARS-CoV-2 Identified by High-Throughput Single-Cell Sequencing of Convalescent Patients B Cells. *Cell* **182**, 73–84, DOI: 10.1016/j.cell.2020.05.025 (2020).

99. Chi, X. *et al.* A neutralizing human antibody binds to the N-terminal domain of the Spike protein of SARS-CoV-2. *Science* eabc6952, DOI: 10.1126/science.abc6952 (2020).

100. Kuo, C. *et al.* RCSB PDB - 7C8R: Complex Structure of SARS-CoV-2 3CL Protease with TG-0203770.

101. Kuo, C. *et al.* RCSB PDB - 7C8T: Complex Structure of SARS-CoV-2 3CL Protease with TG-0205221.

102. Zhang, S., Luan, X. & Wang, F. RCSB PDB - 7C8U: The crystal structure of COVID-19 main protease in complex with GC376.

103. Li, T. *et al.* RCSB PDB - 7C8V: Structure of sybody SR4 in complex with the SARS-CoV-2 S Receptor Binding domain (RBD).

104. Li, T. *et al.* RCSB PDB - 7C8W: Structure of sybody MR17 in complex with the SARS-CoV-2 S receptor-binding domain (RBD).

105. Li, T. *et al.* RCSB PDB - 7CAN: Structure of sybody MR17-K99Y in complex with the SARS-CoV-2 S Receptor-binding domain (RBD).
